# Synergistic Cancer Metabolic Therapy via Co-Delivery of 3‑Bromopyruvate and Temozolomide with a Supramolecular Shuttle

**DOI:** 10.1021/acsami.5c17607

**Published:** 2025-10-22

**Authors:** Rosa Bellavita, Marina Prisco, Sara Palladino, Teresa Barra, Federica Donadio, Emanuela Esposito, Rodolfo Esposito, Giuliana Panico, Jessica Pisano, Paola Venditti, Salvatore Valiante, Annarita Falanga, Gerardino D’Errico, Assunta Lombardi, Stefania Galdiero

**Affiliations:** † Department of Pharmacy, School of Medicine, 9307 University of Naples Federico II, Via Domenico Montesano 49, 80131 Napoli, Italy; ‡ Department of Biology,University of Napoli Federico II, Via Cintia, 80126 Naples, Italy; § Institute of Applied Sciences and Intelligent Systems (ISASI), Naples Cryo Electron Microscopy Laboratory - EYE LAB, National Research Council (CNR), Via Pietro Castellino 111, 80131 Naples, Italy; ∥ Department of Chemical Sciences, University of Napoli Federico II, and CSGI (Unit of Naples), Via Cintia, 80126 Naples, Italy; ⊥ Department of Agricultural Sciences, 9307 University of Naples Federico II, Via Università 100, 80055 Portici, Italy

**Keywords:** self-assembling peptides, glioblastoma, nanofiber, blood−brain barrier, mitochondrial targeting

## Abstract

Combination therapy has shown promise in treating aggressive cancers by using several drugs simultaneously to target different biological pathways, with the added benefit of potentially reducing toxicity. Given the critical role of mitochondrial dysfunction in tumor progression, targeting mitochondrial metabolism represents a promising therapeutic avenue. In this study, we developed a mitochondria-targeted nanofiber based on self-assembling peptides, engineered to co-deliver two complementary therapeutic agents for glioblastoma treatment. The nanofiber carries 1,3-bromopyruvate (BrP), a glycolysis inhibitor, and temozolomide, an alkylating chemotherapeutic, conjugated via a matrix metalloproteinase-9 (MMP-9)-responsive linker for controlled, on-demand release. To enhance selectivity for glioblastoma cells, the nanofiber surface was functionalized with the targeting peptide falGea binding specifically to EGFRvIII, commonly overexpressed in tumor cells, and gH625, a cell-penetrating peptide known to facilitate the blood–brain barrier (BBB) transport. The nanofibers were comprehensively characterized for their aggregation behavior, structural stability, and morphology. Mitochondrial targeting and functional effects were evaluated by using isolated rat brain mitochondria. Therapeutic efficacy was assessed in U-87 MG glioblastoma cells cultured in both 2D and 3D systems. Additionally, BBB permeability was examined by using a dynamic 3D in vitro BBB model, demonstrating the transport-enhancing role of gH625. These findings support the potential of multifunctional, mitochondria-targeted nanofibers as an effective platform for glioblastoma therapy, offering both precision targeting and enhanced drug delivery across the BBB.

## Graphical abstract

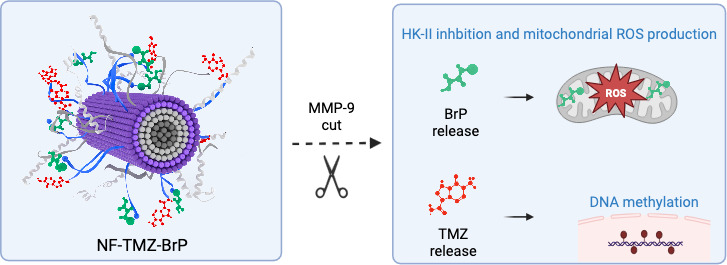

## 1 Introduction

Cancer poses a serious threat to global health due to its diverse causes and complex pathogenesis.^1^ The development of effective and safe drug formulations remains essential for reducing cancer-related morbidity and mortality worldwide.^2^ Despite research advancements that have improved outcomes for many cancer patients, several tumor types still pose significant treatment challenges. Conventional monotherapy often lacks specificity, indiscriminately targeting both malignant and healthy cells and causing severe adverse effects. In contrast, combination therapy, which utilizes multiple drugs, has gained attention for its ability to target multiple molecular pathways while potentially reducing toxicity.^3,4^ In this context, nanotechnology-assisted co-delivery systems offer a promising strategy to improve selective drug delivery and antitumor efficacy.^5^

The efficiency of targeted delivery systems depends on several design parameters including morphology, size, composition, and surface chemistry, as well as the presence of targeting ligands on the surface of nanomaterials.^6^ Among these, the shape of the nanoplatforms is a key determinant to achieve a successful delivery, and nonspherical nanoparticles show a high ability to cross the cell membrane and provide a larger surface area for multifunctionalization.^7^ Besides morphology, particle size also plays a key role, as nanoparticles larger than 500 nm tend to accumulate in the liver and spleen, while those smaller than 5 nm are quickly eliminated by kidneys.^8^ Although both organic and inorganic nanoparticles have been created to cross the blood–brain barrier (BBB) and enter the brain, related brain toxicity makes it difficult to translate inorganic materials into therapeutic applications.^9^ Therefore, biodegradable materials are ideal for controlled and targeted drug delivery, as they can be degraded into small molecules that the body can eliminate or cleanse more readily.^10,11^ In particular, peptide amphiphiles (PAs) represent a particular class of biodegradable materials that spontaneously arrange and form structurally distinct and stabilized nanofibers through their self-assembly.^10–13^ It is also possible to design self-assembly sequences that perform specific additional functions. Thanks to their modularity, the activity of these sequences can be readily tuned by adjusting the quantity and/or type of surface moieties without compromising the integrity of the self-assembled nanostructure. Building on this knowledge, we employed PAs, where the hydrophobic-to-hydrophilic balance regulates self-aggregation behavior, to promote the formation of highly ordered nanofibers.^14^ This makes it possible to precisely regulate how PAs aggregate, producing materials with adjustable characteristics and functionalizing their surfaces with specific moieties.

Among brain cancers, glioblastoma (GBM), one of the most deadly brain tumors, is resistant to treatment due to its communication with the surrounding microenvironment and therapeutic challenges brought on by the presence of the BBB.^15^ While immunotherapies have revolutionized the treatment of several cancers, their efficacy in GBM remains limited because of the tumor’s immunosuppressive environment.^16^ The current standard of care includes surgery followed by radiotherapy and/or temozolomide (TMZ) administration.^17^ TMZ is a DNA alkylating agent widely used in GBM treatment; however, its clinical effectiveness is restricted by a number of issues, including poor water solubility, short plasma half-life, low tumor accumulation, limited selectivity, and off-target toxicity.^18–20^

To address these limitations, this work focuses on enhancing the therapeutic efficacy of the traditional drug TMZ through a multitarget approach aimed at delivering anticancer drugs into mitochondria of cancer cells and targeting tumor bioenergetics, which has recently become an attractive strategy.^21^ In particular, cancer cells exploit the glycolytic pathway, even in an oxygen-rich environment, thus increasing glucose uptake and releasing higher levels of lactate into their microenvironment (Warburg effect). Although glycolysis provides biosynthetic precursors for nucleotide and phospholipid synthesis in rapidly proliferating cells, such as cancer cells, it produces a low amount of ATP, which is essential for cell survival under hypoxic conditions. Thus, cancer cells require glutamine to activate mitochondrial metabolism for the generation of molecules that could support tumor growth (i.e., adenosine triphosphate (ATP), reactive oxygen species (ROS), nicotinamide adenine dinucleotide phosphate (NADPH), amino acids, nucleotides, and lipids).^22,23^ The critical role of mitochondria in the complexity of cancer biology is also attributable to the unlimited cellular proliferative potential, impaired apoptotic cell death, and insensitivity to antigrowth signals, as well as the ability of mitochondria to support tumorigenesis at multiple stages.^24,25^ Targeting and inhibiting glycolysis and mitochondrial metabolism not only disrupts energy production but also helps to overcome the compensatory metabolic adaptations of cancer cells.^26,27^ 1,3- Bromopyruvate (BrP), a halogenated analogue of pyruvate, is an efficient energy blocker inhibiting the enzyme hexokinase II (HK-II), which catalyzes the first step of the glycolytic pathway, whose inhibition can disrupt both mitochondrial and cell metabolism.^28–30^ BrP is effective against a wide range of tumor types including GBM,^31^ where it is implicated in autophagy and cardiolipin degradation, leading to viability loss of cells and inducing a significant increase in oxidative stress with the production of ROS.^32^ Unfortunately, BrP is unable to efficiently cross the BBB, limiting its effectiveness against brain tumors.^33^ To address this challenge, nanotechnology-based strategies need to be explored to develop BrP formulations capable of enhancing its delivery via the bloodstream. Additionally, the complex structural and functional nature of mitochondria hinders selective subcellular targeting, making it difficult to modulate their activity for therapeutic purposes.^29^ Despite these obstacles, mitochondria remain a crucial target for cancer therapy, and effective metabolic reprogramming approaches must simultaneously target both glycolytic and mitochondrial pathways to achieve meaningful therapeutic outcomes.^34–36^

In this study, we employed our self-assembled peptide-based nanofiber vector to simultaneously deliver BrP and TMZ, aiming to selectively target mitochondrial function and disrupt tumor bioenergetics in GBM cell models as a proof of concept. In fact, as TMZ and BrP act on distinct cellular pathways, their co-delivery via a single nanoplatform enables the simultaneous targeting of complementary mechanisms, resulting in a synergistic therapeutic effect.

The crossing of the BBB is achieved through the functionalization of the fiber surface with the cell-penetrating peptide gH625, designed in our laboratory, which was previously demonstrated to fulfill this function.^37–40^ A key issue is the targeting of GBM tumor cells and, more specifically, their mitochondria. To this end, the nanoassembly surface is framed with the peptide falGEA, specifically recognizing the overexpressed epidermal growth factor receptor (both wild type and the mutant variant, EGFRvIII),^41^ while the mitochondrial targeting was achieved by using triphenylphosphonium (TPP^+^), a cationic compound with hydrophobic behavior (Figure 1).^42,43^ Being cationic, TPP^+^ concentrates several hundred-fold into the mitochondria due to its large membrane potential and favors the mitochondrial accumulation of BrP delivered by the fibers. In particular, to enable tumor-specific drug release, BrP was covalently bound to TPP^+^, and this conjugate was attached to the surface of the nanofibers via a matrix metalloproteinase-9 (MMP-9)-responsive linker. This on-demand release strategy takes advantage of the elevated MMP-9 levels typically found in the GBM tumor microenvironment, where several signaling pathways lead to secretion and increase of MMPs in gliomas.^44,45^ Indeed, GBM cells are known to secrete high levels of MMP-9, which plays a critical role in tumor progression, invasion, and metastasis formation by modulating the extracellular matrix.^46^ Once the nanofiber has crossed the cell membrane, elevated levels of MMP-9 cleave the linker, triggering the release of BrP covalently bound to TPP^+^ in the cytoplasm. Guided by the TPP^+^ moiety, BrP is efficiently transported into the mitochondria, where it can exert its therapeutic effects.

**Figure 1:**
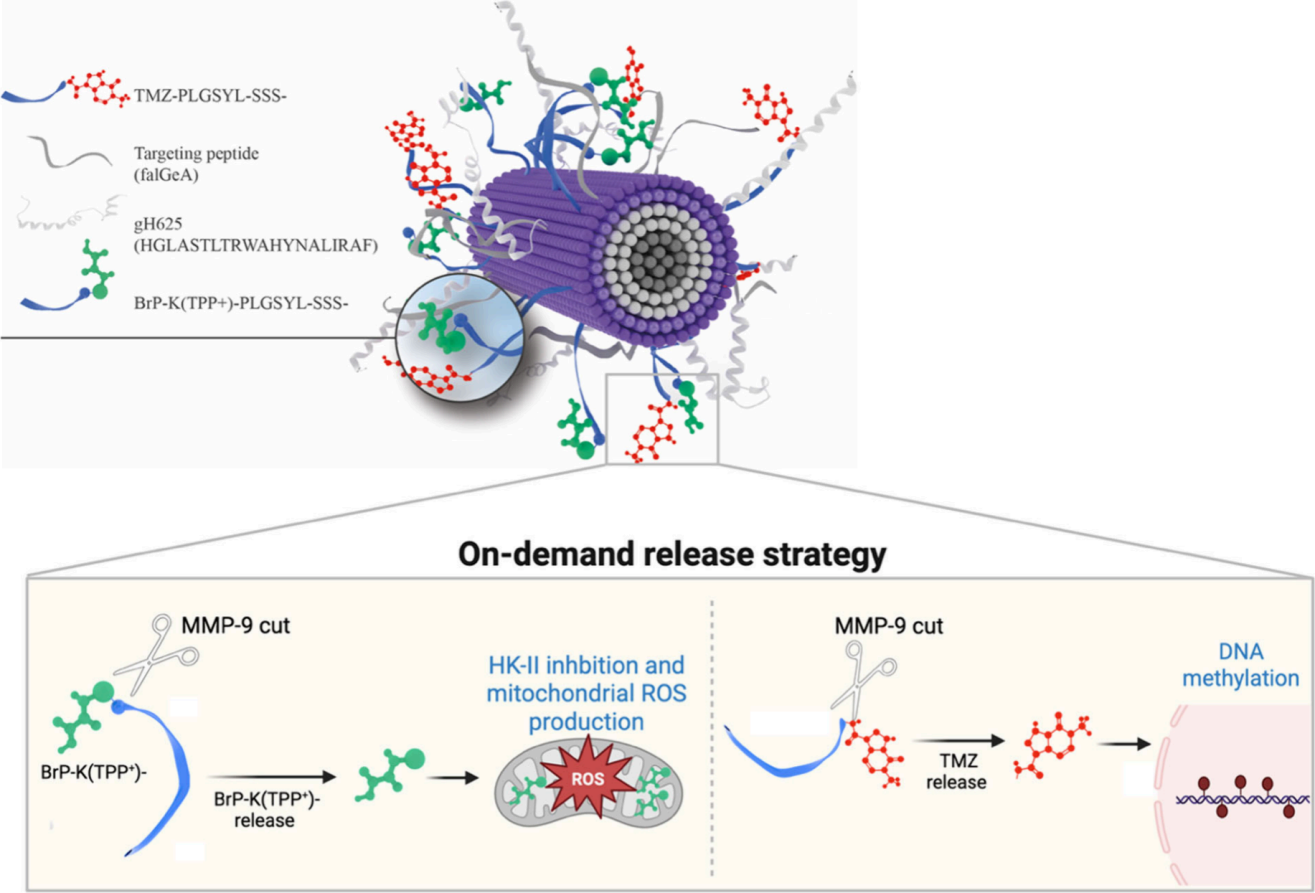
Representation of mitochondria-targeted nanofiber NF-TMZ-BrP and the hypothetical mechanism of the release of BrP and TMZ by the MMP-9 proteolytic cut. The figure was created partially with BioRender.

The mitochondrial-targeted nanofiber, obtained from the coassembly of PAs, was described for aggregation behavior and structural stability using a combination of fluorescence microscopy, circular dichroism, and electron paramagnetic resonance (EPR) spectroscopy. The ability of the BrP conjugated on the nanofiber to inhibit mitochondrial respiration was evaluated on mitochondria isolated from rat brain, and the production of ROS during mitochondrial respiration was directly monitored by EPR.

In addition, to enhance the therapeutic effect toward GBM, the mitochondrial-targeted nanofiber was further functionalized with TMZ using the same MMP-9-responsive release strategy previously shown to be effective in our recent work.^10^ We assessed the cytotoxic effect of BrP alone and in combination with TMZ in in vitro experiments on healthy cells and GBM cell lines (U-87 MG, ATCC) in both 2D and 3D cultures. In addition, the ability of the nanofiber functionalized with BrP alone and in combination with TMZ to penetrate the BBB was investigated in a dynamic 3D in vitro BBB model.

In conclusion, this work reports the design and structural/functional characterization of a nanoplatform that selectively targets brain cancer cells, improves the BBB transfer efficiency, and enhances the efficacy of loaded drugs. Its ability to respond to real-time environmental cues, such as tumor-specific enzyme activity, enables more personalized and adaptive treatment approaches. These unique features hold the potential to improve the treatment of brain cancers, reducing side effects, and enhancing therapeutic outcomes.

## 2 Results and Discussions

### 2.1 Design and Synthesis of Mitochondria-Directed Nanofibers for Enhanced Brain Delivery

The design strategy plays a crucial role in enabling the development of self-assembling materials with integrated surface functionalities. We designed and developed a mitochondria-targeted platform based on our previous knowledge on PAs that spontaneously form supramolecular nanofibers (NFs) upon dispersion in aqueous solution.^10,13,47^ We aimed to expand the functionality of our supramolecular nanostructures by incorporating multiple peptide-based components, designed to coassemble in a controlled manner, in order to efficiently and selectively enter cells and deliver different drugs. The nanoplatform structure consists of two peptides P1 and P2, each featuring a hexaalanine sequence and a lipidic tail (C19) conjugated to the ε amino group of a terminal lysine. These hydrophobic units drive the self-assembly process, forming the core of the nanofiber, stabilized by the hydrophobic interactions between PAs. The inclusion of two charged residues with oppositely charged side chains (negative in P1 and positive in P2) provides additional intermolecular ionic bonding leading to catanionic mixed aggregates.^13^ Additionally, the N-terminus of the structural peptide P2 can be covalently functionalized with several moieties intended for display on the nanocarrier surface. This modular design allows for the surface decoration of the nanofiber with specific bioactive molecules. In particular, focusing on aggregates tailored for GBM treatment, the nanofiber is formulated with PAs conjugated with i) the cell-penetrating peptide gH625 that crosses cell membranes including the BBB, as evidenced in vitro and in vivo;^39,48,49^ ii) the targeting peptide falGea binding the specific receptor overexpressed in the tumor site;^41^ iii) the drugs (i.e., BrP or TMZ) where therapeutic agents are linked via an MMP-9-sensitive sequence enabling the enzyme-triggered release in the tumor site; iv) the mitochondrial targeting moiety, triphenylphosphonium (TPP^+^), attached covalently to the peptide carrying BrP. Following the internalization of the nanofiber into the cytosol, the TPP^+^–BrP conjugate is liberated via proteolytic cleavage. Once released, TPP^+^ facilitates the selective accumulation of BrP within the mitochondria, driven by the organelle’s high membrane potential. All of these peptides self-assemble into nanofibers through distinct steps shown in Figure 2.

**Figure 2:**
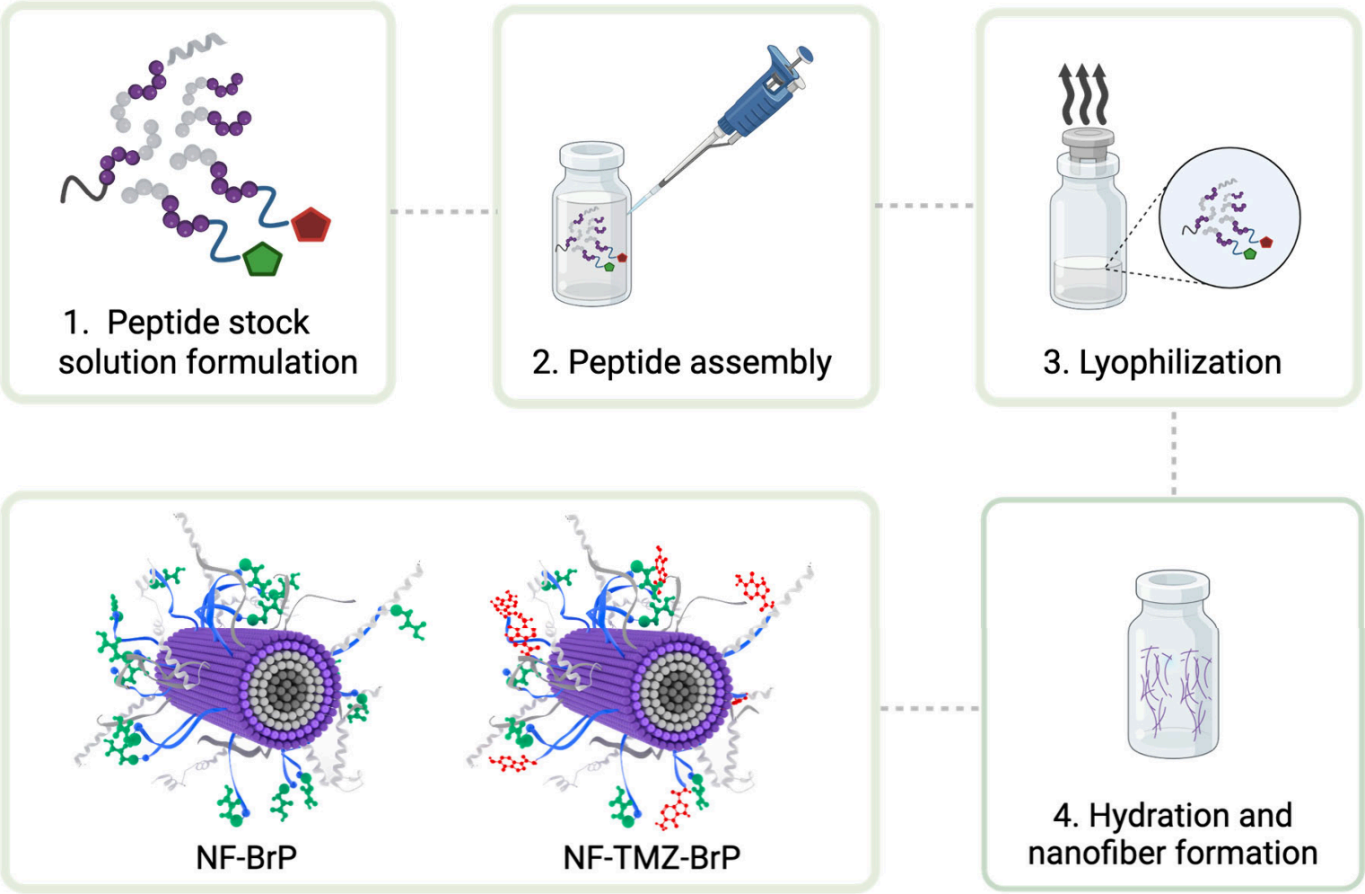
Illustration of the nanofiber formation process for NF-BrP and NF-TMZ-BrP. This figure was created with BioRender.

The SPPS methodology combined with the Fmoc/*t*-Bu strategy was used for the peptide synthesis. Each peptide’s C-terminus was bound to the C19; in particular, a lysine residue protected with an Mtt group on its side chain was selected as the first amino acid, as the Mtt protecting group can be selectively removed under mild acid conditions, facilitating the subsequent conjugation of the C19. The drugs TMZ and BrP were covalently attached at the N-terminus after the MMP-9-cleavable sequence (PLGSYL), following an on-demand release strategy. For BrP a lysine residue was also added at the N-terminus; thus, the BrP was coupled at the main chain amino terminus, while the mitochondrial targeting moiety TPP^+^ was incorporated on the side chain of the lysine, performing the coupling between the carboxylic acid on TPP^+^ and free amine deprotected from the Mtt group. All peptides were cleaved from the resin along with protecting groups, purified by HPLC, and characterized by ESI-MS.

### 2.2 Engineering of Mitochondria-Targeted Nanofibers

Our previous studies demonstrated that peptide amphiphiles P1, P2, P3, and P2-t exhibit a strong propensity to self-assemble when mixed at the precise molar ratio of 1:0.74:0.2:0.06.^10^ This optimized composition led to the obtainment of NFs with a characteristic length of 160 ± 40 nm and a diameter of 11 ± 3 nm.^10^ This nanofiber formulation was specifically tailored for GBM targeting and showed a significant ability to cross the BBB due to the presence of the peptide gH625 (included in P3). When it was further functionalized with TMZ (10 μM), we observed a remarkable cytotoxic effect against GBM cell lines U-118 and U-87.^10^ In this study, the NF was adapted to engineer the mitochondria-targeted formulation NF-TMZ-BrP carrying the drugs BrP and TMZ and the mitochondrial-targeting moiety TPP^+^, which facilitates the selective delivery of BrP into the mitochondria. The initial step consists of quantifying the optimal surface density of each functional moiety on the NF structure. We first formulated NFs with different BrP loadings (5%, 10%, and 15%). Cytotoxicity screening revealed that the 5% BrP formulation (final concentration of 5 μM) offered the best balance between therapeutic efficacy and minimized the formation of large aggregates in the cellular environment (Figure S1). Once the optimal BrP concentration was established, the nanofiber surface was further functionalized with P2-TMZ, a peptide–drug conjugate carrying TMZ, resulting in the NF-TMZ-BrP formulation used for combination therapy studies. The mitochondria-targeted nanofibers NF-BrP and NF-TMZ-BrP were characterized in terms of aggregation, morphology, and structure (Figure 3). The critical aggregation concentration (CAC) is essential, as nanofiber (NF) formation does not occur when peptides are mixed below this threshold. In contrast, coassembly above the CAC promotes interactions between hydrophobic and hydrophilic domains, driving NF formation. This information is therefore crucial for establishing the appropriate experimental conditions. We calculated the CAC by coassembling the peptides at the defined ratio. We obtained a CAC value of 16.9 ± 0.1 μM coassembling the peptides P1, P2, P2-t, P3, and P2-BrP at the ratio 1:0.64:0.06:0.2:0.1, and the analysis of the zeta potential indicated a positively charged surface for NF-BrP due to the presence of TPP^+^, measuring a value of +39.5 ± 0.9 mV (Table 1).

**Table 1:** Summary of CAC and Zeta Potential Parameters for the Nanoparticle Formulations NF, NF-BrP, and NF-TMZ-BrP

**Formulation**	**Composition**	**CAC (μM)**	**Zeta potential (mV)**
NF	P1+P2+P2-t+P3 (1:0.74:0.06:0.2)	14.2 ± 0.1	+4.4 ± 1.2
NF-BrP	P1+P2+P2-t+P3+P2-BrP (1:0.64:0.06:0.2:0.1)	16.9 ± 0.1	+39.5 ± 0.9
NF-TMZ-BrP	P1+P2+P2-t+P3+P2-BrP+P2-TMZ (1:0.54:0.06:0.2:0.1:0.1)	6.7 ± 0.1	+34.4 ± 1.9

**Figure 3:**
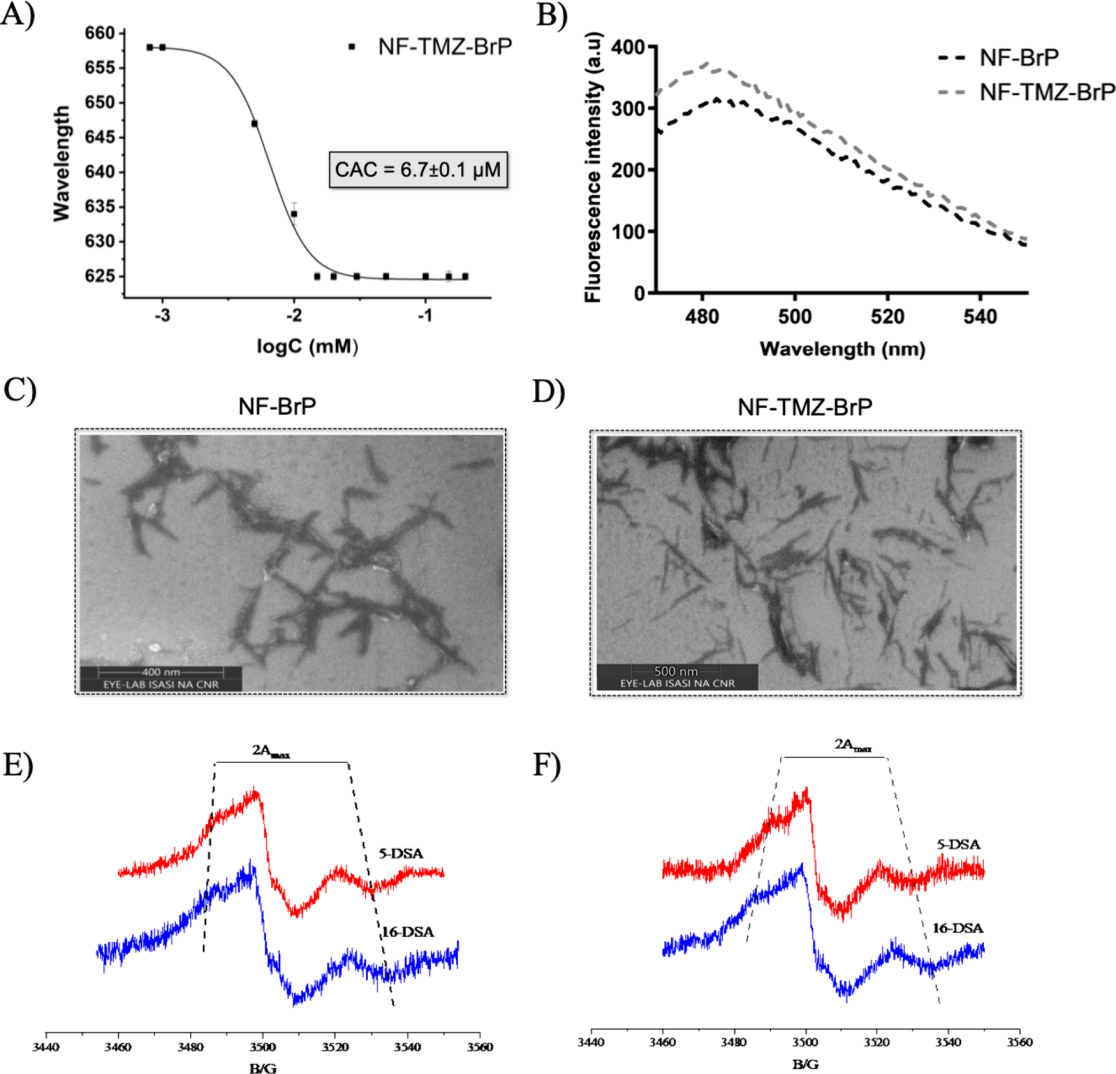
Panel A: The CAC value was calculated by plotting the wavelength that corresponded to the maximal fluorescence emission of Nile red as a function of the nanofiber NF-TMZ-BrP concentration. Data are presented as the mean ± standard deviation (SD) from three independent experiments. Panel B: ThT spectra were recorded for each NF formulation. Panels C and D: SEM images of NF-BrP (C) and NF-TMZ-BrP (D). Panels E and F: EPR spectra of 5-DSA (red) and 16-DSA (blue) in NF-BrP (panel E) and NF-TMZ-BrP (panel F).

When we further included the nanofiber composition P2-TMZ (5%), we observed a stronger propensity to aggregate, obtaining a lower CAC value, 6.7 ± 0.1 μM (Figure 3A), and the zeta potential was still positive (+34.4 ± 1.9 mV). Interestingly, as reported in Table 1, the CAC of NF made by coassembling P1, P2, P3, and P2-t (1:0.74:0.06:0.2) is 14.2 ± 0.1 μM,^10^ indicating that the addition of P2-BrP induces only a slight increase of the CAC, while the addition of TMZ favors the aggregation process.

The NF assembly and formation were also supported and investigated by a thioflavin (ThT) assay as highlighted in Figure 3B. Although this dye is commonly used to track the development of β-sheet amyloid fibrils, it can be exploited to investigate the generation of self-assembled nanostructures. Increased fluorescence results from its interaction with these structures, which limits rotation between the benzene and benzothiazole rings. After the NF hydration and ThT addition, we recorded an enhancement of ThT emission at approximately 480 nm for NF-TMZ-BrP decorated with both drugs on its surface. Furthermore, SEM analysis confirmed the obtainment of NFs with a variable length of 120–250 nm and diameter of 14–25 nm (Figure 3C and 3D). These dimensions fall within the optimal range for drug delivery applications.

Additionally, each nanofiber, NF-BrP and NF-TMZ-BrP, was characterized by electron-paramagnetic resonance (EPR) spectroscopy (Figures 3E and 3F). Previous EPR characterization of the NF inner core formed by the hydrophobic tails revealed a compact molecular organization that significantly restricts rotational mobility.^10^ Inclusion of peptides functionalized with bulky moieties, such as the targeting peptide and TMZ, in the nanofiber formulation was found to increase the tail mobility, with the effect being more pronounced for the outer chain segments than for the inner ones. This is due to the steric hindrance between the targeting peptide and/or the drug exposed on the fiber that propagates to the inner core and disturbs its structuring and is not necessarily correlated to the CAC values, which more directly depend on the hydrophobic effect.^10^ The results reported in this work show that the increase in chain segment mobility is further enhanced when the P2-BrP peptide is included in the NF (Figure 3E and 3F). In particular, the TPP^+^ group is expected to exert significant steric repulsion, thus making the peptide self-assembly slightly looser. This is true both in the absence of P2-TMZ (NF-BrP, Figure 3E, 2Amax values equal to 44 and 52 G observed for 5 and 16 DSA, respectively) and in its presence (NF-TMZ-BrP, Figure 3F, 2Amax values equal to 41 and 52 G observed for 5 and 16 DSA, respectively). Nevertheless, it should be noted that the values observed for 16-DSA remain higher than those observed for liposomes or similar lipid assemblies, indicating a more compact and ordered organization of the inner segments of the hydrophobic tails.^50^

### 2.3 Structural Stability of Mitochondria-Targeted Nanofibers

The stability of the secondary structure of the peptides composing the mitochondria-targeted NFs was investigated by circular dichroism (CD) spectroscopy. The nanofiber NF-BrP presented good stability under different environments. As evidenced by CD spectra in Figure 4, the nanofiber NF-BrP adopted a β-type conformation with the minimum at 220 nm, which is preserved under the dilution effect (panel A), ionic strength (panel C), and pH environments (panel E).

**Figure 4:**
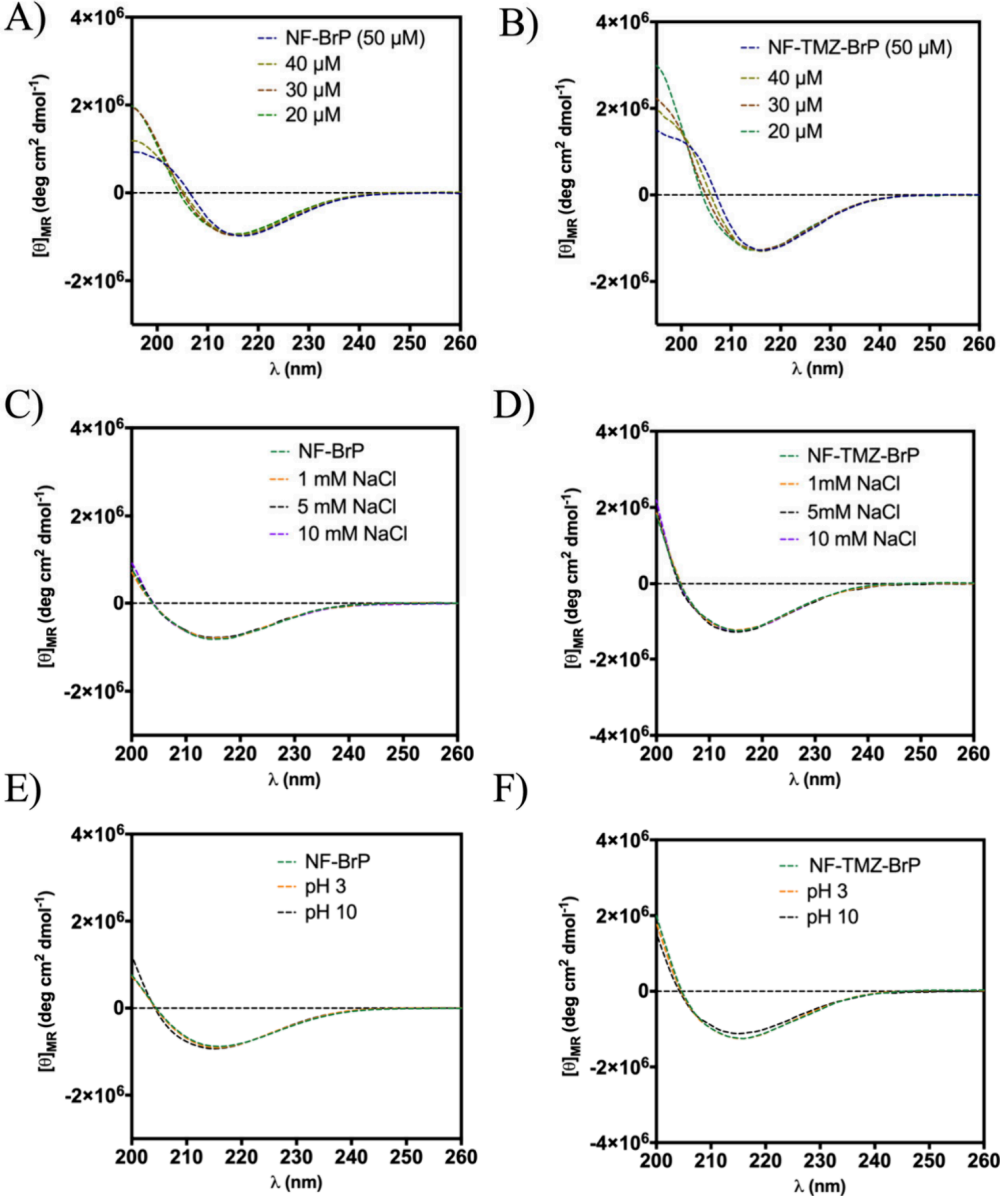
Panels A, C, and E report the CD spectra of NF-BrP under the dilution effect (A), ionic strength (C), and pH environments (E). Panels B, D, and F report the CD spectra of NF-TMZ-BrP under the dilution effect (B), ionic strength (D), and pH environments (F).

Similar physicochemical properties were observed for the multifunctionalized nanofiber NF-TMZ-BrP. The addition of the drug TMZ (5 μM) on the nanofiber surface did not induce significant changes in both secondary structure and morphology (Figures 4B, 4D, and 4F). In fact, NF-TMZ-BrP presented a good stability under dilution, pH, and ionic strength changes, preserving its β-sheet conformation.

### 2.4 Mitochondria-Targeted Nanofibers for Efficient Cell Membrane Crossing

The cell-penetrating peptide gH625 is a membranotropic peptide that can promote the cellular internalization of different nanosystems, as already demonstrated in our previous studies.^10,12,47^ This process occurs without causing membrane disruption and is mainly driven by a translocation mechanism involving localized and transient membrane destabilization, followed by reorganization. In the present work, we confirmed the exposure and the capacity of gH625, when covalently linked to PAs and presented on the NF surface, to induce membrane fusion using large unilamellar vesicles (LUVs) made of PC:Chol (1:1) as a model of eukaryotic membranes.

To confirm proper surface exposure of the peptide on the NFs, we conducted a tryptophan quenching assay in aqueous solution using acrylamide as the quencher to target the tryptophan residue present in gH625. The experiment was performed on control NFs functionalized with P3 and P2-t, as well as on NF-BrP and NF-TMZ-BrP, to assess whether drug loading induces steric hindrance, affecting gH625 accessibility. Upon the addition of increasing concentrations of acrylamide (0.02–0.22), the tryptophan fluorescence was progressively quenched, resulting in a concentration-dependent decrease in emission intensity. The extent of tryptophan accessibility in the different NF formulations was quantified by plotting the quenching data and calculating the Stern–Volmer constant (*K*_sv_) via linear regression (Figure 5A). The *K*_sv_ values, 4.4 ± 0.1 for NF, 3.1 ± 0.2 for NF-BrP, and 6.2 ± 0.3 for NF-TMZ-BrP, are within the same range, suggesting that the Trp remains accessible to acrylamide, even when the nanofiber surface is loaded with both drugs.

**Figure 5:**
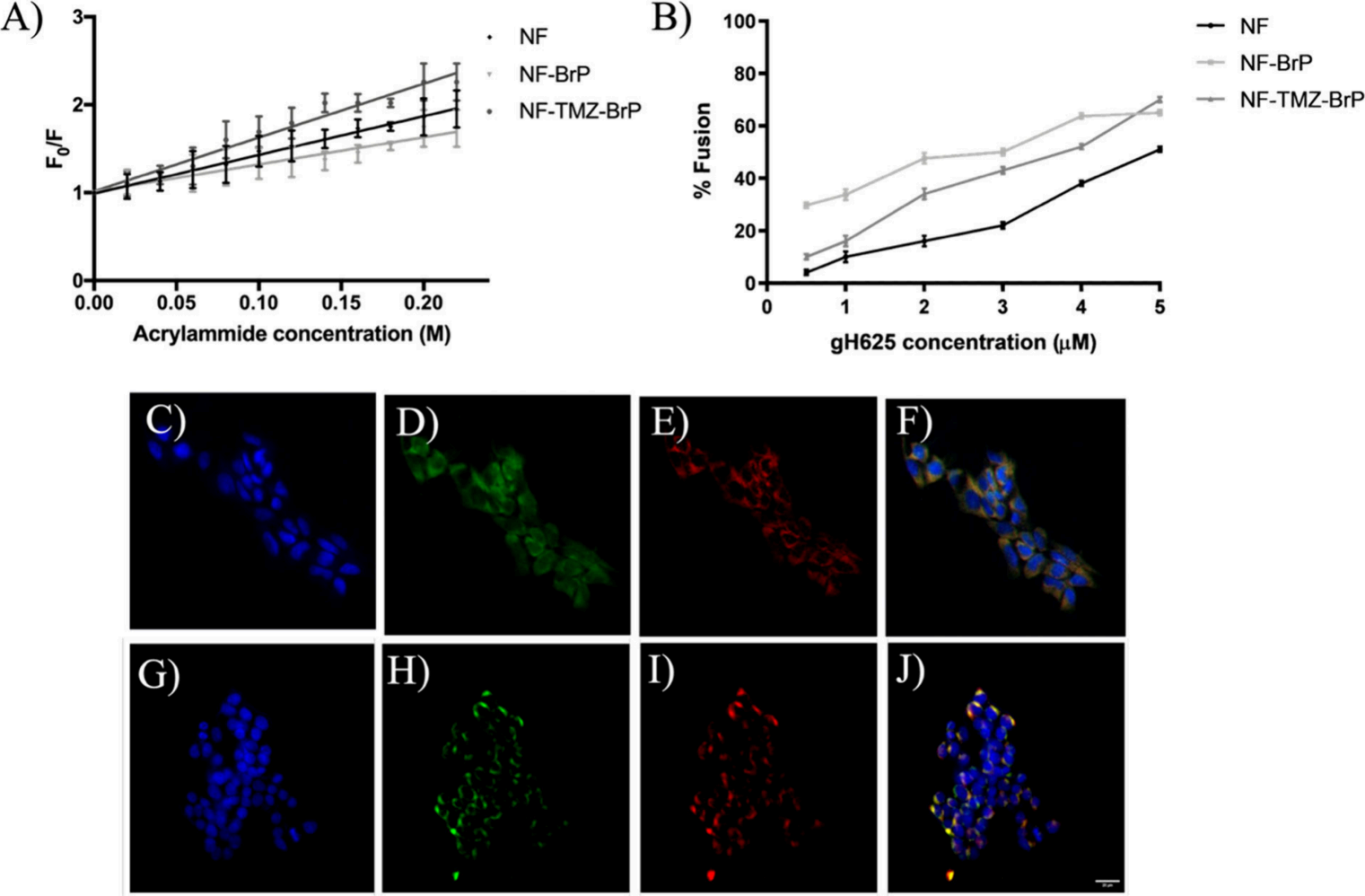
Panels A and B report the data analyzed with the Stern–Volmer equation and the percentage of fusion ability of gH625 placed on NF, NF-BrP, and NF-TMZ-BrP, respectively. The experiments were performed in triplicate, and data represent the mean ± SD. Panels C–J report the U87 cell uptake of FITC-NF-TMZ-BrP (D) and NF-TPP^+^-FITC (H) after 90 min of incubation, followed by MitoTracker 300 nM treatment and staining with Hoechst. Panels C and G: nuclei stained with Hoechst; D and H: FITC localized inside the cells; E and I: MitoTracker localization in mitochondria; F and J: merging of the three channels, MitoTracker signal colocalizes with the nanofiber. Bar = 20 μm.

The fusogenic activity of the peptide gH625 exposed on the NF surface was monitored by performing the lipid mixing assay in the presence of a population of LUVs (PC:Chol = 1:1) labeled with -NBD and -Rho, used as acceptor and donor of fluorescence energy transfer, and unlabeled LUVs (PC:Chol, 1:1). Nanofibers NF, NF-BrP, and NF-TMZ-BrP were prepared with 10% of peptide P3 carrying gH625 and were added at the concentrations of 0.5, 1, 2, 3, 4, and 5 μM. A significant membrane fusion mediated by gH625 was observed upon titration of the LUVs with both NF-BrP and NF-TMZ-BrP. In particular, at the highest gH625 concentration (5 μM), approximately 70% membrane fusion was detected for both formulations (Figure 5B). This evidence confirms that gH625 is properly exposed on the NF surface and retains its fusogenic functionality, which is correlated to its ability to cross membrane bilayers.^50^

These results were further validated in vitro using U-87 cells as a GBM model. To evaluate the ability of the NFs to cross the cell membrane, we prepared two fluorescently labeled formulations: (i) FITC-labeled NF-TMZ-BrP (FITC-NF-TMZ-BrP), where 6% of the P2 peptide was substituted with P2-f to track cellular internalization and overall NF localization; and (ii) NF-TPP^+^-FITC, in which P2-BrP was replaced by FITC-TPP^+^-P2 (5%) to specifically assess mitochondrial localization of BrP following MMP-9-mediated cleavage. Intracellular distribution of both NF formulations was analyzed by fluorescence microscopy, with nuclei and mitochondria counterstained using Hoechst and MitoTracker, respectively.

As shown in Figure 5, after 90 min of treatment, FITC-NF-TMZ-BrP was clearly observed inside the cells as green fluorescence (Figure 5D), demonstrating colocalization with the red fluorescent MitoTracker (Figures 5E and 5F). Similarly, mitochondrial colocalization was observed for the short peptide fragment TPP^+^-FITC (Figures 5I and 5J), released from NF-TPP^+^-FITC via proteolytic cleavage by MMP-9 expressed in U-87 cells (Figure S14). These findings suggest that the NFs are internalized by U-87 cells and exhibit colocalization with mitochondria, although the certainty that NF entered the mitochondria can only be given to us by future electron microscopy investigations. Moreover, the observed mitochondrial colocalization of TPP^+^-FITC further supports that the TPP^+^ targeting moiety promotes accumulation of the released BrP within mitochondria, likely driven by their high membrane potential.

### 2.5 Biocompatibility and Controlled Release of Drugs via an On-Demand Strategy

We validated the biocompatibility and the MMP-9 responsive drug release from the NF using healthy brain endothelial cells (HBMEC) that constitutively express low levels of MMP-9, in contrast to U-87 cells, where MMP-9 is highly expressed (Figure S2).

First, we assessed the cytotoxicity of NF-BrP (BrP 5, mM) and NF-TMZ-BrP (TMZ and BrP, 5 μM) on HBMEC cells in the absence of exogenous MMP-9. Previous studies demonstrated that naked NFs (without TMZ and BrP) exhibited excellent biocompatibility, showing no impact on cell viability up to 72 h in both GBM and HBMEC cells.^10^ Here, we show that treatment with NF-BrP and NF-TMZ-BrP similarly did not cause significant changes in HBMEC viability over 72 h, with values comparable to untreated controls (Figure 6A), confirming the safety of our NFs for healthy cells.

**Figure 6:**
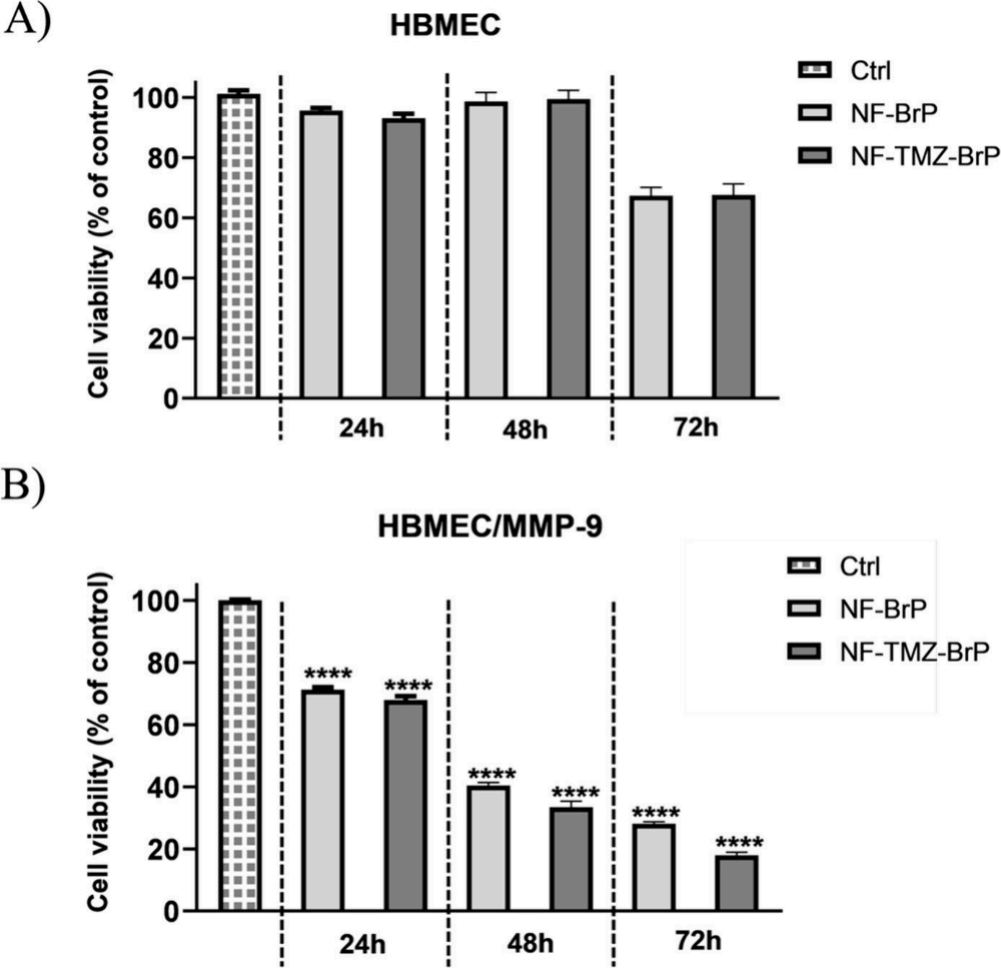
HBMEC cell viability assay. Panel A: Cells were treated with NF-BrP (BrP, 5 μM) and NF-TMZ-BrP (TMZ and BrP, 5 μM) for 24, 48, and 72 h. Panel B: Cells treated with NF-BrP (BrP, 5 μM) and NF-TMZ-BrP (TMZ and BrP, 5 μM) incubated previously with MMP-9. The cell viability was measured for 24, 48, and 72 h. Cell viability is expressed as a percentage relative to untreated control cells (Ctrl). Data are presented as means ± SEM from triplicate analyses. ****p < 0.0001.

The on-demand release strategy employed here has previously demonstrated effective in delivering TMZ conjugated to NFs in GBM cells, with drug release triggered by MMP-9-mediated proteolytic cleavage.^10^

To validate our MMP-9-responsive delivery system, HBMEC cells were treated with NF-BrP and NF-TMZ-BrP preincubated with exogenous MMP-9 to ensure complete drug release. Under these conditions, cell viability significantly decreased, showing an approximately 80% reduction after 72 h for both formulations (Figure 6B), compared to only about 20% reduction without MMP-9. Indeed, already at 48 h we observe a significant reduction of cell viability (60%). These results support our hypothesis that the cytotoxic effects of NF-BrP and NF-TMZ-BrP are mediated by MMP-9-triggered drug release, enabling selective activity in environments with elevated MMP-9 levels, such as the tumor microenvironment.

### 2.6 Translocation of Nanofibers across the BBB

Given the limited ability of BrP and TMZ to cross the BBB and the challenges in achieving selective targeting, we functionalized our nanocarrier with the delivery peptide gH625 to enhance BBB permeability and facilitate targeted brain delivery, thereby minimizing off-target effects. In our previous work, we developed an in vitro dynamic BBB model to assess the translocation efficiency of our NFs.^10^ This model features a coculture of HBMEC and human pericytes from placenta (hPC-PL) cells seeded on a porous membrane within the LB2 bioreactor. The LB2 is a specialized cell culture chamber designed to support the formation of physiological barriers, including the BBB, featuring dual compartments, an upper (UP) and a lower (LC) chamber, separated by a biocompatible, low-protein-binding porous membrane.^10,37,38^ This setup is integrated with a Liveflow peristaltic pump that enables continuous circulation of nutrients and metabolites, closely mimicking in vivo conditions.^10^ Here, to quantitatively evaluate NF translocation across the BBB, we performed a spectrofluorimetric assay on the solution collected from the LB2 bioreactor, measuring the fluorescence of FITC-labeled NF-BrP and NF-TMZ-BrP, in which a fraction of peptide P2 was replaced with P2-f (Figures 7A–7D).

**Figure 7:**
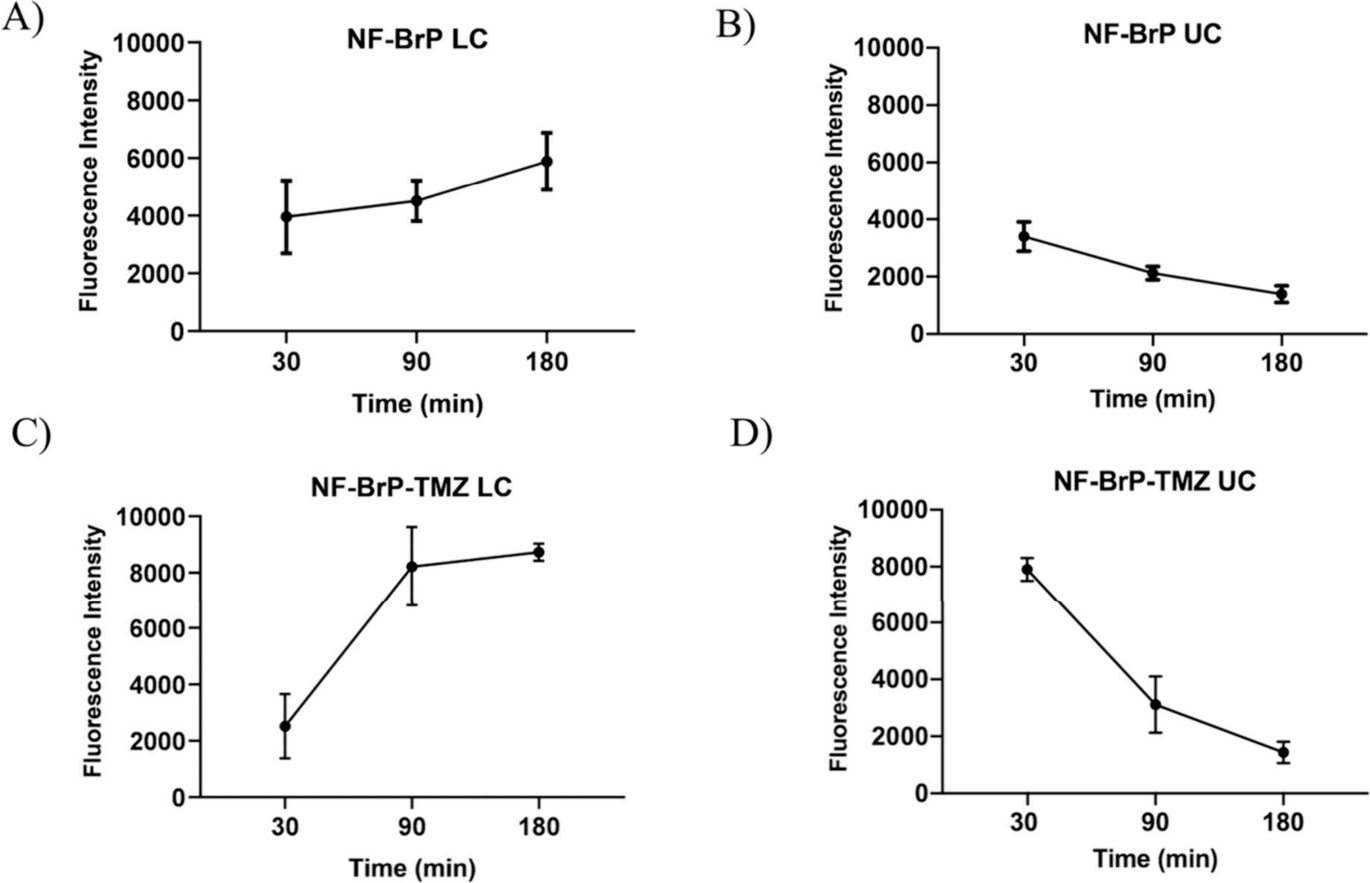
Spectrofluorometric assay of NF-BrP (A, B) or NF-TMZ-BrP (C, D) with a BBB dynamic in vitro model. The passage outside the upper (UC) and lower chambers (LR) was evaluated with medium samples. Results are reported as means ± SEM of a triplicate analysis.

### 2.7 NF-BrP Impact on Cytotoxicity and Mitochondrial Function in Vitro

#### 2.7.1 Assessment of BrP-Mediated Cytotoxic Effects

To evaluate the antiproliferative effects of BrP delivered via NF-BrP (5 μM), we first assessed its impact on 2D and 3D U-87 cells and compared the results to those obtained with free BrP (Figures 8A and 8B). As shown in Figure 8A, treatment with NF-BrP for 24 h did not significantly affect U-87 cell viability; however, a 30% reduction was observed at 48 h, which further increased to approximately 40% after 72 h (Figure 8A). In contrast, free BrP exhibited minimal cytotoxicity, with a decrease in cell viability only observed at high concentrations (50–150 μM) after 24 h (see Figure S3), 48 h, and 72 h (Figure 8C and 8D). Notably, even at 150 μM, free BrP reduced cell viability by no more than 20% and only after prolonged exposure. These findings indicate that BrP, when conjugated to the nanofiber surface, exhibits significantly enhanced cytotoxicity compared to that of its free form, even at a much lower concentration (5 μM). This improved efficacy is likely attributable to the enhanced intracellular delivery provided by the NF, which facilitates the access of BrP to its intracellular target sites.

**Figure 8:**
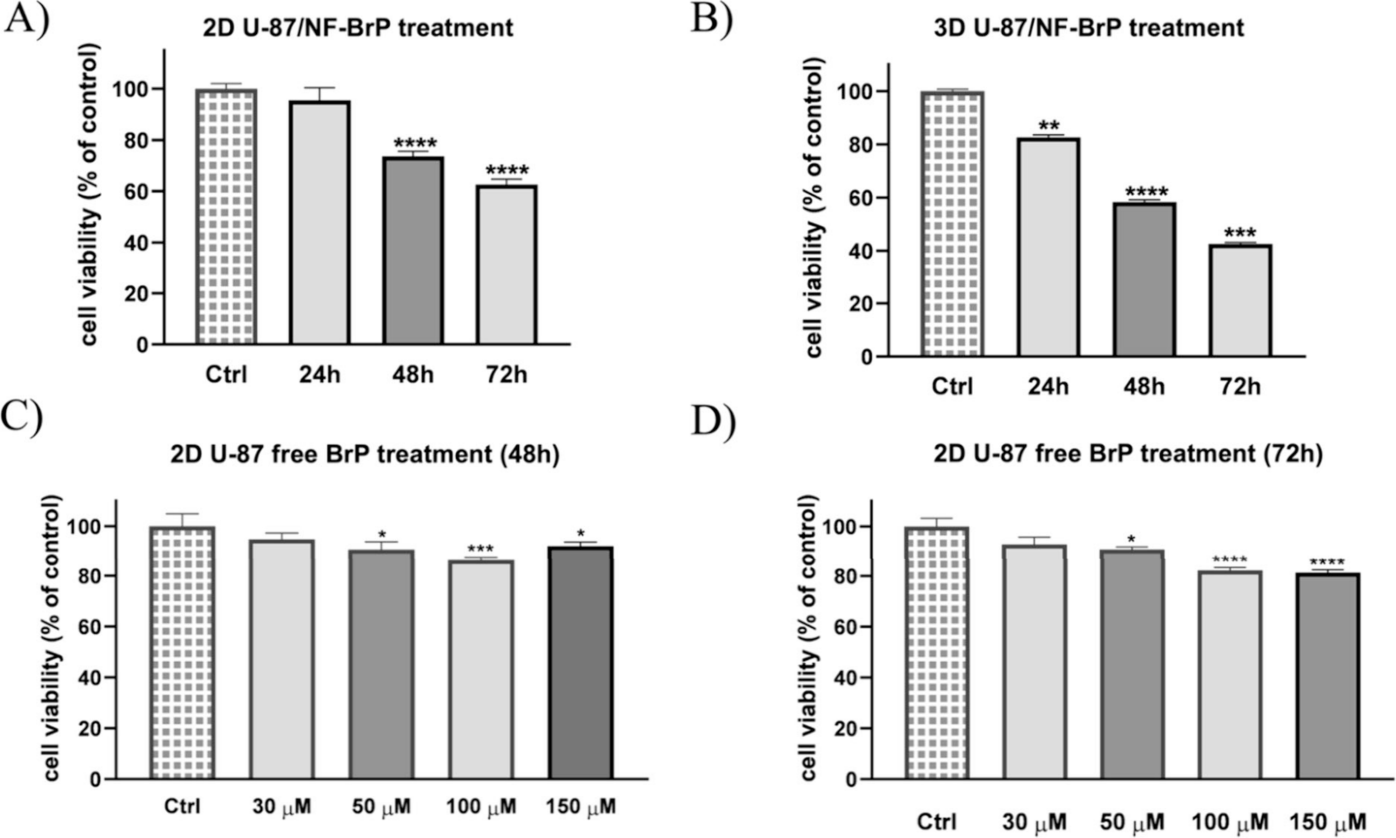
U-87 cell viability assay. Panel A: Cell viability after NF-BrP (BrP, 5 μM) treatment on 2D U-87 for 24, 48, and 72 h. Panels B and C: U87 cells were treated with free BrP at different concentrations at 48 and 72 h. Panel D: Cell viability after NF-BrP (BrP, 5 μM) treatment on 3D U-87 for 24, 48, and 72 h. Cell viability is expressed as a percentage of untreated control cells (Ctrl). Data are presented as means ± SEM from triplicate analysis: *p < 0.1; **p < 0.01; ****p < 0.0001.

To better replicate the in vivo tumor microenvironment, we also conducted cytotoxicity experiments using 3D U-87 spheroids. These spheroids were cultured in the lower chamber of the LB2 bioreactor, and the cytotoxic effect of NF-BrP (BrP, 5 μM) was evaluated by following its translocation across the BBB model. NF-BrP was administered once daily for three consecutive days. After 72 h, we observed a significant reduction (∼55%) in tumor cell viability compared to the untreated control (Figure 8B). These results strongly support our hypothesis that the NFs are able to cross the BBB and deliver the drug effectively to the tumor site. Moreover, the observed cytotoxicity at a relatively low drug concentration highlights the potential of this approach to minimize damage to healthy tissue by avoiding large systemic doses.

#### 2.7.2 Impact of BrP, NF, and NF-BrP on Mitochondrial Function in Vitro

The direct in vitro interaction of free BrP, NF (without BrP), or NF-BrP with mitochondria could potentially affect their functionality. To investigate this issue, we isolated mitochondria from rat brain and preincubated them with the various formulations in the respiratory chamber. The preincubation lasted 8 min, after which mitochondrial respiratory substrates and adenosine diphosphate (ADP) were sequentially added to the respiration medium. We employed two different respiratory substrates, i.e., succinate (+rotenone) and pyruvate (+ malate), since they provide us information on respiratory pathways linked to mitochondrial respiratory complexes I and II, respectively.

As observed in Figure 9, at the concentration of 5 μM, BrP induced a significant inhibition of respiration detected in the presence of ADP (about 50%) when using succinate + rotenone (Figure 9A) or pyruvate + malate (Figure 9B) as substrate. The inhibitory effect of BrP is concentration-dependent, and a dose–response relationship is reported in the Supporting Information (Figure S4). Considering that when measured in the presence of ADP, the control of mitochondrial respiration is shared by the overall reactions involved in the antioxidation of substrates, and the activity of reaction implicated in the synthesis and export of ATP, our data indicate that BrP compromises the ability of mitochondria to produce ATP and confirm the known toxicity exerted by BrP on mitochondria functionality.

**Figure 9:**
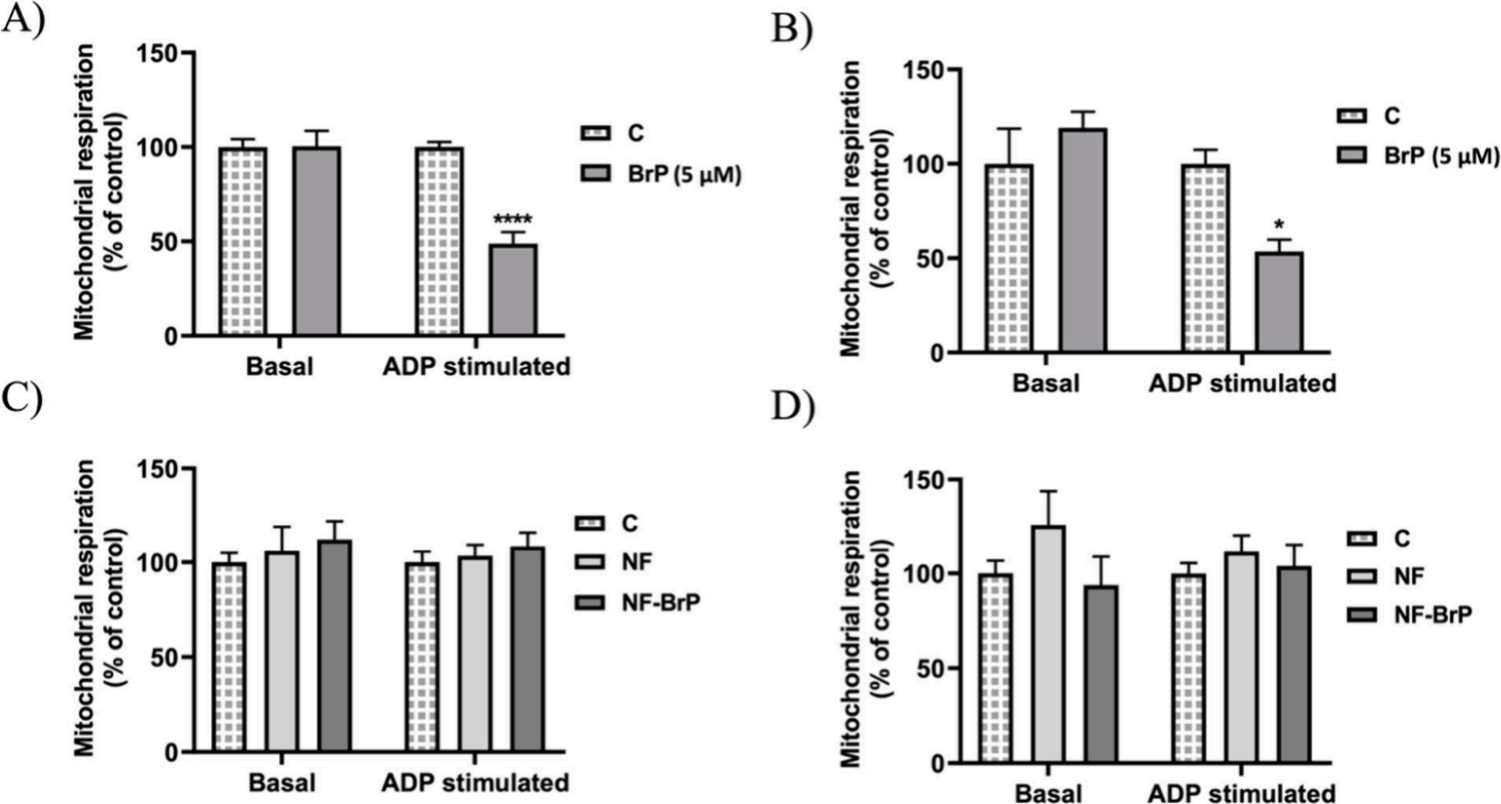
In vitro effect of BrP, NF, and NF-BrP on mitochondrial respiration. Panels A and B: Mitochondrial respiration was detected after the treatment with BrP in the presence of succinate+rotenone (panel A) or pyruvate+malate (panel B) as respiratory substrates. Panels C and D: Mitochondrial respiration was detected after the treatment with NF-BrP in the presence of succinate+rotenone (panel C) or pyruvate+malate (panel D) as respiratory substrates. Respiration was detected in the absence (basal) or the presence of ADP (ADP stimulated). % of oxygen consumption variation is reported. Data ± SEM; ****p < 0.0001; *p < 0.05.

NF and NF-BrP did not significantly impact mitochondrial respiration, regardless of the respiratory substrate used (Figures 9C and 9D), indicating that direct exposure to the nanofibers does not impair mitochondrial function. Furthermore, BrP conjugated to the nanofiber surface did not inhibit mitochondrial respiration, suggesting that its activity is dependent on release via MMP-9 cleavage, an event that predominantly occurs in cancer cells with an elevated level of MMP-9 expression.

Additionally, since BrP is involved in ROS generation, the formation of these species was directly monitored by EPR spectroscopy during mitochondrial respiration and following treatment with BrP or the NF formulations. The experiments were performed in parallel to those described above. After 8 min of preincubation, succinate was added as a mitochondrial respiratory substrate. In addition, the spin probe mito-TEMPO (Figure 10A) was added to all samples at a concentration of 10 μM. Mito-TEMPO carries a triphenylphosphonium moiety, which leads to its rapid solubilization in functional mitochondria where the TEMPO (tetramethylpiperidinyl-oxyl) radical is quenched to diamagnetic species by the ROS naturally produced during respiration. Therefore, the intensity of the TEMPO EPR signal, a triplet due to the coupling of the unpaired electron with the nitrogen nucleus, decreases (Figure 10B). The slope of this decay (relative intensity vs time) is a quantitative index of physiological ROS production during mitochondrial respiration, and its flattening indicates an increase in their production (Figure 10C).

**Figure 10:**
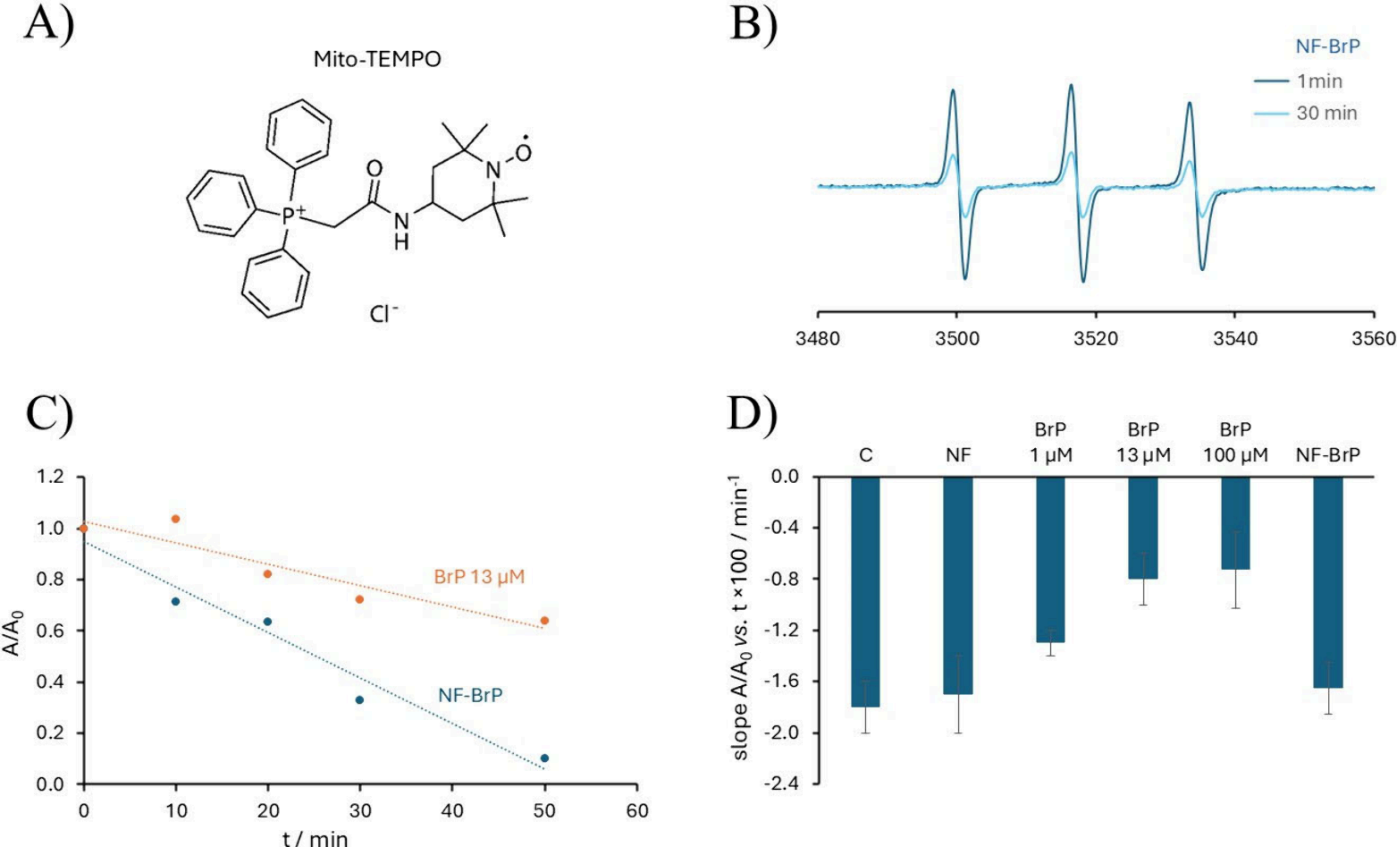
Panel A shows the molecular structure of the spin probe mito-TEMPO. Panel B shows the EPR spectra of Mito-TEMPO incorporated into mitochondria in the presence of NF-BrP, recorded shortly after the addition of succinate as respiratory substrate and after 30 min. Panel C shows the decay with time of the signal intensity (obtained by double integration of the registered spectrum) of Mito-TEMPO in mitochondria in the presence of free BrP and NF-BrP. Panel D compares the slope of mito-TEMPO decay in respiring mitochondria in the presence of the different systems indicated.

Inspection of Figure 10D shows that NF-BrP did not affect mitochondrial ROS production, as the observed slope is almost identical to that observed in the absence of any vector. On the other hand, BrP reduces the slope in a dose-dependent manner, indicating its ability to induce ROS production, which contributed to its toxic effect. However, when BrP is bound to the fiber, no effect is observed. These results converge with those reported above, showing that for BrP to exert its toxic effects, it must be released from the fiber, thus providing the opportunity for responsive drug release operated by specific metalloproteases.

### 2.8 Synergistic Effect of Drug Combination on U-87 Cells

Combining drugs with different mechanisms of action targeting distinct cellular pathways can help overcome resistance and produce a synergistic effect, thereby enhancing the overall anticancer efficacy. The combined cytotoxic effect of the nanofiber NF-TMZ-BrP, cofunctionalized with BrP and TMZ (both at 5 μM), was evaluated in 2D and 3D-U87 cells at 24, 48, and 72 h. As shown in Figure 11A, no significant reduction in 2D cell viability was observed after 24 h; however, an already significant decrease (∼30%) was detected at 48 h. After 72 h, the cytotoxic effect became more pronounced, with approximately 60% reduction in cell viability. We already observed a 20% reduction in 3D U-87 cells after 24 h of NF-TMZ-BrP treatment, which increases to ∼70% after 72 h (Figure 11B). This response was significantly greater than that observed with free BrP alone, and it also exceeded the cytotoxic effect of free TMZ, which previously demonstrated only a 40% reduction in cell viability at its highest tested concentration (250 μM) after 72 h.^10^ These results suggest that the co-delivery of BrP and TMZ (both at a concentration of 5 μM) via the same nanocarrier significantly enhances antiproliferative efficacy, likely due to synergistic interactions and improved intracellular delivery.

**Figure 11:**
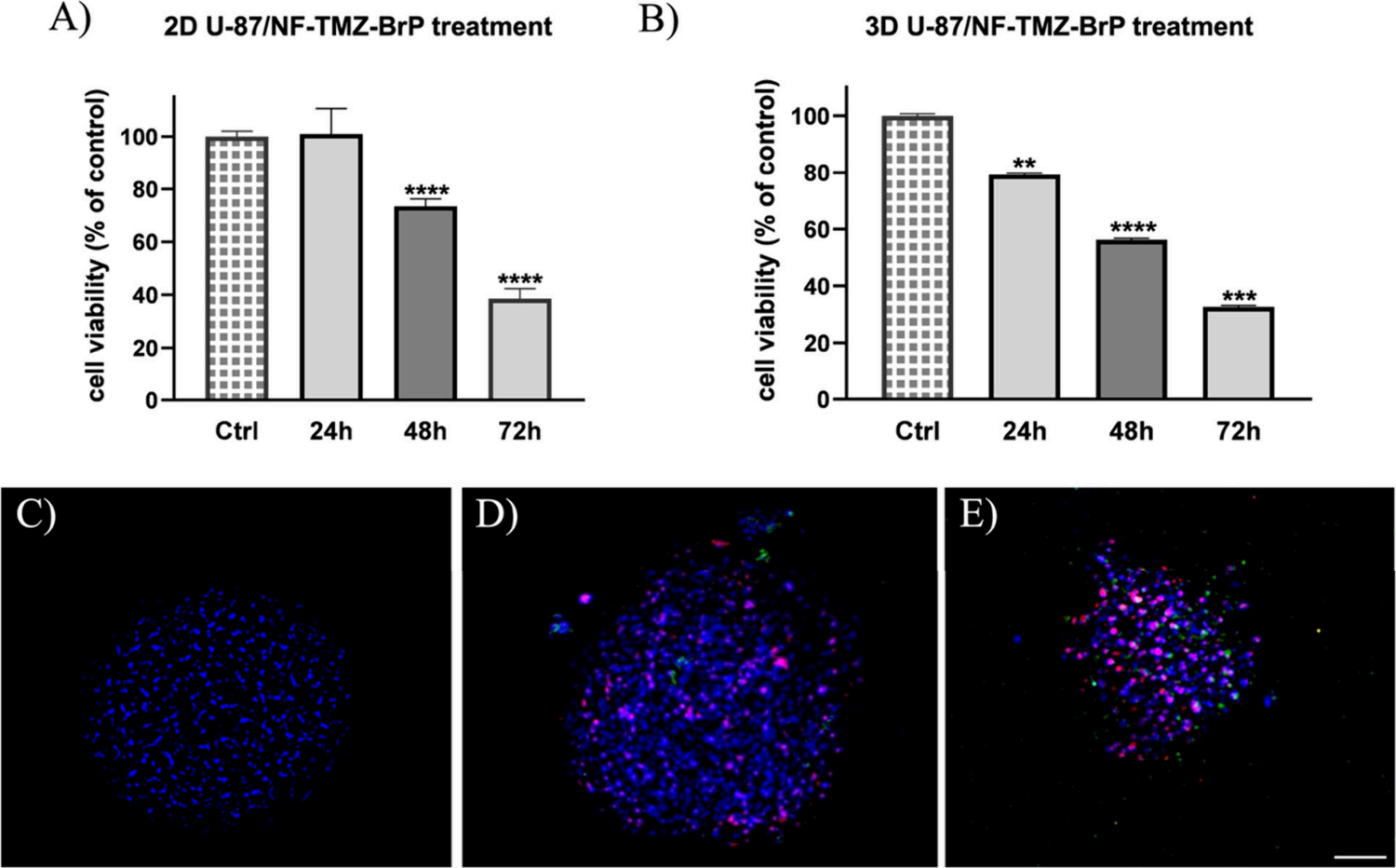
Panels A and B report cell viability measured for NF-TMZ-BrP (TMZ and BrP, 5 μM) on 2D and 3D U-87, respectively, after 24, 48, and 72 h. Panels C–E: Representative images of Annexin V and PI staining on 3D U-87 (C) and after treatment with NF-BrP (D) and NF-TMZ-BrP (E). All spheroids were labeled with DAPI. Scale bar = 500 mm.

In addition, we investigated the mechanisms (necrosis and apoptosis pathways) underlying cell death induced by BrP and TMZ released from nanofibers by performing Annexin V/PI assays on 3D U-87 spheroids following treatment with NF-BrP and NF-TMZ-BrP. As shown in Figure 11, both NF-BrP-treated cells (Figure 11B) and NF-TMZ-BrP-treated cells (Figure 11C) displayed a substantial presence of necrotic cells (RFP channel: *l*_ex_: 550 nm, *l*_em_: 650 nm) and apoptotic cells (GFP channel: *l*_ex_: 395 nm, *l*_em_: 475 nm), in contrast to the untreated control (Figure 11A), where only nuclear staining (DAPI: *l*_ex_: 358 nm, *l*_em_: 461 nm) was observed. These findings indicate that the observed cytotoxic effects in 3D spheroids involve both necrotic and apoptotic pathways. This dual mechanism is likely due to the combined action of BrP, which disrupts glycolysis and mitochondrial ATP production, and TMZ, which induces DNA methylation and subsequent double-strand breaks by interfering with DNA repair processes. Given the high glycolytic dependency of GBM cells, ATP depletion by BrP may sensitize the cells to TMZ-induced genotoxic stress, thereby enhancing the overall therapeutic efficacy.

Moreover, significant morphological alterations were observed in spheroids following treatment with NF-BrP and NF-TMZ-BrP. In particular, the morphological analysis of treated spheroids revealed a consistent reduction in spheroid surface area compared to appropriate controls (Figure 12A). To determine whether this decrease was due solely to a reduction in size or also involved cell disaggregation from spheroids, we performed a time course analysis of the perimeter-to-roundness ratio (Figure 12B–12D).

**Figure 12:**
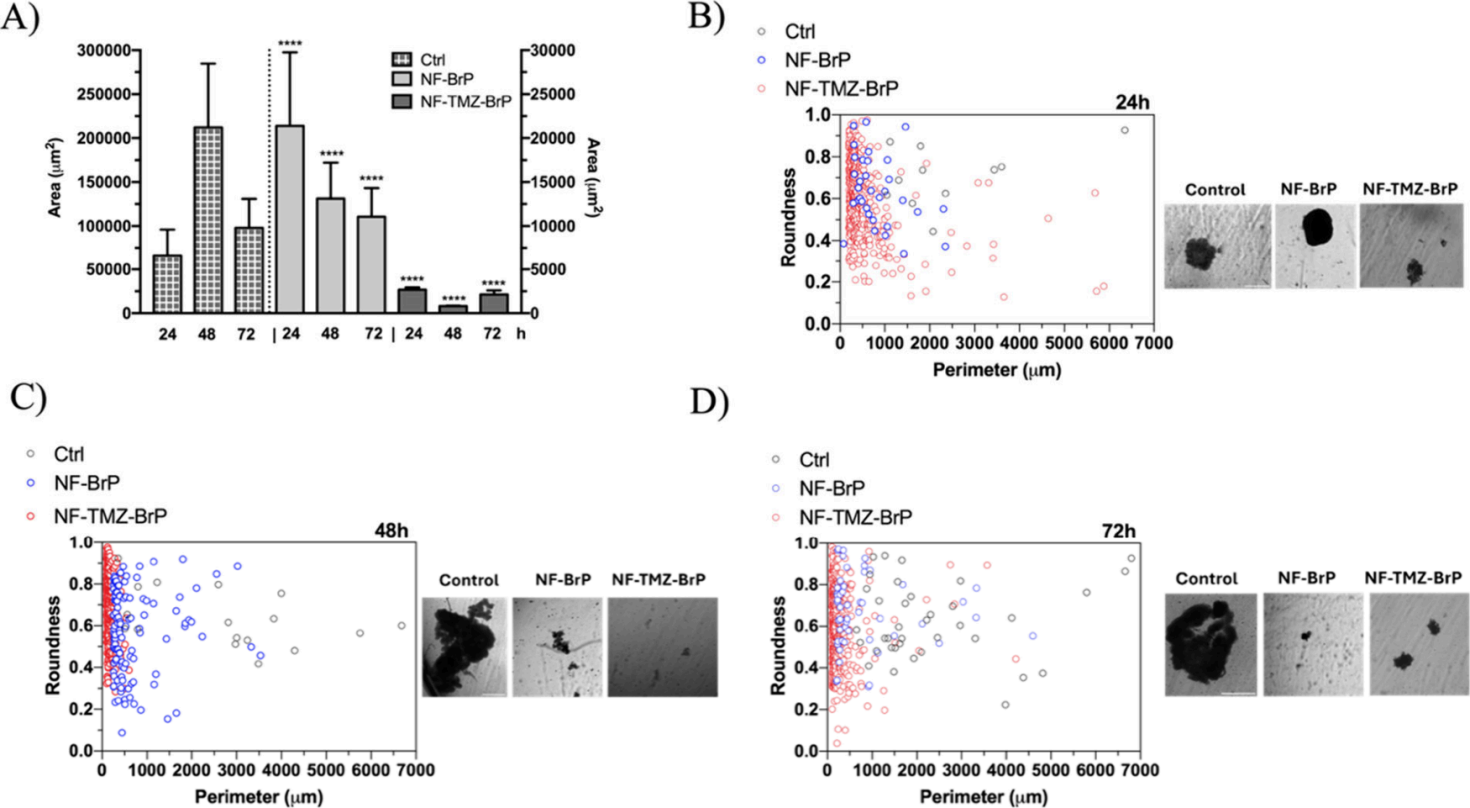
Time-dependent effect of NF-Br and NF-TMZ-BrP on spheroid parameters. Panel A reports surface area evaluation of 24, 48, and 72 h. Control (Ctrl) and NF-BrP- and NF-TMZ-BrP-treated spheroids. Histograms on the right side of the dotted line are on the right axis. ****p < 0.001 compared with time-correspondent control. Panels B–D report morphological parameter evaluation on control (Ctrl) and NF-BrP-treated and NF-BrP-TMZ-treated spheroids. Ratio between the perimeter and roundness of control (black), NF-BrP-treated (blue), and NF-TMZ-BrP-treated (orange) spheroid population through time (B: 24, C: 48, and D: 72 h). For each time point representative images of spheroids are provided. Scale bars correspond to 500 μm.

The analysis rationale is based on the fact that roundness serves as an indicator of the morphological integrity of the entire spheroid as well as of individual cells or cell clusters detaching from the main spheroid body. In parallel, the perimeter reflects the overall spheroid or cell group size. After 24 h of treatment, a marked difference in the perimeter-to-roundness ratio was observed between treated and control spheroids. Control spheroids maintained a relatively high mean ratio (∼3390), indicative of an intact, compact morphology. In contrast, NF-BrP-treated spheroids exhibited a significantly lower mean ratio (∼1278), with an even more pronounced reduction in NF-TMZ-BrP-treated spheroids (∼668), suggesting early morphological disruption (Figure 12B). After 48 h, both perimeter and roundness values of the NF-BrP and NF-BrP-TMZ treated spheroids began to diverge from those of the controls, which continued to grow with stable morphology. The treated spheroids showed increasing disaggregation into smaller, less cohesive structures, with a loss of spheroid-like shape (Figure 12C). By 72 h, this trend was more pronounced. Treated spheroids further disassembled into small units, likely single cells or small clusters, as evidenced by increased roundness and decreased perimeter. The corresponding perimeter/roundness ratios dropped significantly to ∼256 for NF-BrP-treated and ∼111 for NF-TMZ-BrP-treated spheroids, compared to ∼3775 in controls (Figure 12D). These results confirm that the treatments induced progressive spheroid disaggregation, supporting the cytotoxic and structurally disruptive effects of the NF formulations over time.

## 3 Conclusions

Co-delivery strategies are particularly advantageous for treating aggressive cancers, where conventional monotherapies often fail due to resistance and poor drug penetration. Building on previous work,^10^ we optimized a self-assembling NF for GBM therapy, adapted for mitochondria-targeted combination treatment. The NF was cofunctionalized with BrP and TMZ and incorporated the mitochondrial-targeting moiety TPP^+^ along with the BBB-penetrating peptide gH625. Comprehensive structural and physicochemical characterization, including CAC determination, zeta potential analysis, EPR spectroscopy, thioflavin T fluorescence, and SEM, confirmed successful NF assembly, efficient drug incorporation, preserved morphology, and a compact internal structure suitable for drug delivery. Both NF-BrP and NF-TMZ-BrP retained stable β-sheet conformations across a range of dilutions, ionic strengths, and pH values. The addition of TMZ did not alter NF structure or integrity, underscoring the platform’s robustness under physiological conditions.

To address the poor BBB permeability of BrP and TMZ, the NF surface was functionalized with gH625. This peptide retained its fusogenic and internalization properties and remained surface-accessible post-drug loading. In a dynamic in vitro BBB model, NFs demonstrated time-dependent translocation across the barrier, confirming efficient BBB penetration. Fluorescence microscopy in U-87 GBM cells further confirmed intracellular NF uptake and mitochondrial colocalization, indicating gH625-mediated internalization and TPP^+^-driven mitochondrial targeting of BrP upon release.

Developing biodegradable, biocompatible drug delivery systems remains a key goal of current research, and our cofunctionalized NFs showed no cytotoxicity toward healthy cells, lacking elevated MMP-9 expression. In contrast, MMP-9 pretreatment triggered drug release and led to a ∼80% reduction in cell viability after 72 h, validating the NF’s MMP-9-responsive, tumor-specific release mechanism.

The mitochondrial effects and antiproliferative activity of BrP delivered via NF-BrP (5 μM) were evaluated in both 2D and 3D U-87 GBM models and compared to free BrP. NF-BrP significantly reduced cell viability, while free BrP induced minimal cytotoxicity only at concentrations >50 μM and prolonged exposure, indicating that NF-mediated delivery enhances intracellular uptake and therapeutic efficacy at concentrations at least 30-fold lower, thereby supporting also reduced systemic toxicity. Moreover, unlike free BrP, NF and NF-BrP did not impair mitochondrial respiration and showed no effect on ROS production, suggesting that BrP toxicity requires release, likely via MMP-9 cleavage in cancer cells.

To enhance efficacy through multidrug delivery, we evaluated NF-TMZ-BrP (5 μM of each drug) in 2D and 3D U-87 models. This co-delivery led to a pronounced reduction in viability, surpassing the effects of either free drug alone. These results suggest a synergistic enhancement through improved cellular delivery and combined action on distinct cellular pathways.

Following NF-BrP and NF-TMZ-BrP treatment, we observed substantial apoptosis and necrosis, likely due to BrP-mediated inhibition of glycolysis and mitochondrial ATP synthesis, sensitizing GBM cells to TMZ-induced DNA methylation and double-strand breaks. Given GBM reliance on glycolysis, ATP depletion enhances susceptibility to genotoxic stress, thus amplifying the therapeutic impact. This was further supported by marked morphological changes in 3D spheroids, including reduced surface area and a drop in the perimeter-to-roundness ratio, where roundness reflects structural integrity and perimeter denotes size, indicative of progressive spheroid disassembly. These structural changes corroborate the potent cytotoxic activity and tumor-disruptive potential of the NFs.

Our findings pave the way for future studies focused on evaluating the long-term efficacy and safety of the nanocarrier system in vivo. Data obtained from animal models will be essential to validate the translational potential of this platform and support its development for clinical application. We recognize that the current lack of in vivo data represents a limitation and an opportunity for future exploration.

In summary, we developed a multifunctional peptide-based nanocarrier designed for mitochondria-targeted, MMP-9-responsive, and BBB-permeable drug delivery, demonstrating a strong potential for effective and selective GBM treatment. Our findings highlight how nanotechnology can enable combinatorial therapy by co-delivering synergistic agents, thereby enhancing therapeutic efficacy while minimizing systemic toxicity. Future research should focus on optimizing nanocarrier design for the coencapsulation of multiple therapeutic agents, with particular emphasis on maintaining structural stability and achieving controlled, sustained release. In parallel, a deeper investigation into the molecular mechanisms underlying drug synergy will be essential to tailor combination therapies to specific tumor profiles. Collectively, this study positions our peptide-based NFs as a promising platform for co-delivery strategies in oncology. With further rational design, these biocompatible nanocarriers could be adapted to incorporate diverse therapeutic cargoes, opening avenues for more effective combination treatments. Ultimately, we envision that such NFs can serve as versatile tools for precision cancer theranostics and can be extended to address a broad spectrum of pathologies.

## 4 Materials and Methods

### 4.1 Materials

#### 4.1.1 Materials for Synthesis

Fmoc-protected amino acids were purchased from GL Biochem Ltd. (Shanghai, China). The Rink amide *p*-methylbenzhydrylamine (MBHA) resin, pure oxyma, 1-[bis­(dimethylamino)­methylene]-1*H*-1,2,3-triazolo­[4,5-*b*]­pyridinium 3-oxide hexafluorophosphate (HATU), [2-[2-(Fmoc-amino)­ethoxy]­ethoxy]­acetic acid (Fmoc-PEG2-OH), trifluoroacetic acid (TFA), and piperidine were acquired from Iris-Biotec GmbH. Fmoc-Lys­(Mtt), 1,1,1,3,3,3-hexafluoro-2-propanol (HFIP), 1-hydroxybenzotriazole hydrate (HOBt), *N*,*N*,*N*′,*N*′-tetramethyl-*O*-(1*H*-benzotriazol-1-yl)­uronium hexafluorophosphate, *O*-(benzotriazol-1-yl)-*N*,*N*,*N*′,*N*′-tetramethyluronium hexafluorophosphate (HBTU), nonadecanoic acid (C19), *N,N′*-diisopropylcarbodiimide (DIC), *N*-(3-(dimethylamino)­propyl)-*N*′-ethylcarbodiimide (EDC), *N*-hydroxysuccinimide (NHS), triisopropylsilane (TIS), matrix metalloproteinase-9 (MMP-9), Nile Red, thioflavin T, *N,N*-diisopropylethylamine (DIEA), 3-methyl-4-oxo-3,4-dihydroimidazo­[5,1-*d*]^1–3,5^tetrazine-8-carboxylic acid (temozolomide acid), 3-bromopyruvic acid (BrP), (2-carboxyethyl)­triphenyl­phosphonium bromide, 5(6)-carboxyfluorescein (Fam), 1-ethyl-3-(3-(dimethylamino)­propyl)­carbodiimide (EDC), *N*-hydroxysuccinimide (NHS), 4-dimethylaminopyridine (DMAP), 5- and 16-DOXYL stearic acid (5- and 16-DSA), and (2-(2,2,6,6-tetramethyl­piperidin-1-oxyl-4-ylamino)-2-oxoethyl)­triphenyl­phosphonium chloride (Mito-TEMPO) were purchased from Merck (Milan, Italy). *N,N*-Dimethylformamide (DMF), dichloromethane (DCM), diethyl ether (Et_2_O), water, and acetonitrile (MeCN) were acquired from commercial sources (Merck and VWR), were of reagent grade, and were utilized without additional purification. Phospholipids including phosphatidylcholine (PC), cholesterol (Chol), rhodamine, and 12-(*N*-methyl-*N*-(7-nitrobenz-2-oxa-1,3-diazol-4-yl)) (NBD)-phosphatidyl­ethanolamine (Rho-PE and NBD-PE, respectively) were purchased from Avanti Polar Lipids (Birmingham, AL, USA).

#### 4.1.2 Materials for Cell Culture

Dulbecco’s modified Eagle medium (DMEM) culture medium, fetal bovine serum (FBS), penicillin/streptomycin (P/S, 10000 U/ml), l/glutammine (L/GLUT, 200 mM), trypsin-EDTA, phosphate buffer saline (PBS), 4′,6-diamidino-2-phenylindole (DAPI), and Lucifer yellow assay were acquired from Merck (Milan, Italy). The chamber slide was bought from Sarstedt (Milan, Italy). Mito Tracker Deep Red, PrestoBlue assay, and Hoechst were purchased from ThermoFisher Scientific (Waltham, MA, USA). Annexin VI/PI was purchased from Elabscience (Florida, USA). Livebox2 (LB2) and the peristaltic pump (Liveflow) were bought from IVTech (Pisa, Italy), and porous membranes PET 25 mm and 0.45 μm were purchased from it4ip (Louvain-la-Neuve, Belgium). Human brain microvascular endothelial cells from human healthy brain (HBMEC), endothelial cell growth supplement (ECGS, 1%), and fibronectin solution (2 μg/cm^2^) were purchased from Innoprot (Bizkaia, Spain), while human pericytes from placenta (hPC-PL) cells and SupplementMix low serum base were purchased from Promocell (Heidelberg, Germany). Ultralow-attachment 6-well plates were from Corning (New York, USA).

### 4.2 Peptide Synthesis

#### 4.2.1 Structural Peptide Synthesis

The structural peptides P1 [NH2-GDDS-AAAAAA-K­(C19)] and P2 [NH2-GKRS-AAAAAA-K­(C19)] with hydrophobic and hydrophilic units were obtained using the solid-phase peptide synthesis (SPPS) technique as reported elsewhere.^10,12^ The amino acid Fmoc-Lys­(Mtt)-OH was used as the first building block to attach on the resin Rink amide MBHA (100–200 mesh) after Fmoc deprotection employing a basic solution of 20% piperidine in DMF. After the attachment of the first amino acid, the six-alanine tail was added through repeated cycles of coupling reactions and Fmoc removals. Each coupling reaction was achieved through two cycles under ultrasound irradiation. In particular, the resin was treated for 10 min with Fmoc-AA (3 equiv), HBTU (3 equiv), HOBt (3 equiv), and DIPEA (6 equiv) in DMF under ultrasound for the initial coupling reaction. During the second step, the supernatant was discarded, and the reaction procedure was repeated twice. Fmoc removal was performed with a solution of 20% piperidine in DMF (2 × 5 min) under ultrasound. Then, the hydrophilic unit including -Gly-Asp-Asp-Ser (in P1) and -Gly-Lys-Arg-Ser- (in P2) was linked to the Ala6 sequence by the same procedure described above. After the accomplishment of the linear sequence, the Mtt group was orthogonally removed from the C-terminus lysine side chain with the mild acid mixture (TFA:TIS:DCM, 1:5:94, v:v:v) via reiterated cycles (10 times) for 25 min. Mtt group removal was confirmed using the colorimetric Kaiser test and high-performance liquid chromatography (HPLC), following an acetylation test performed on a small resin sample. Then, the coupling of nonadecanoic acid (2 equiv) was implemented with HATU (2 equiv) and DIPEA (4 equiv) in NMP for 2 h under stirring.

#### 4.2.2 Functional Peptide Synthesis

The functional peptides P3, P2-t, P2-TMZ, and P2-BrP were designed to bear the specific moiety covalently linked to the structural peptide P2 (Table 2). The peptide P3 bearing cell-penetrating peptide gH625 was featured by the sequence HGLASTLTRW­AHYNALIRAF linked to the peptide P2 through repeated cycles of coupling reactions and Fmoc removals using the SPPS protocol described above. The same SPPS procedure was used to synthesize the peptide P2-t bearing the targeting peptide falGea, which was characterized by all d-amino acids. Both for peptides P3 and P2-t, after the Mtt deprotection in mild acid conditions, the conjugation of lipid tail C19 was carried out in the presence of HATU (2 equiv) and DIPEA (4 equiv) in NMP for 2 h at room temperature (rt).

**Table 2:** Peptide Sequences Involved in Nanofiber Formation

**Peptide**	**Sequence**
**P1**	GDDS-AAAAAA-K(C19)
**P2**	GKRS-AAAAAA-K(C19)
**P3** (delivery peptide = gH625)	HGLASTLTRWAHYNALIRAF-GKRS-AAAAAA-K(C19)
**P2-t** (targeting peptide)	falGea-SSS-GKRS-AAAAAA-K(C19)
**P2-f** (labeled peptide)	FITC-PEG2-GKRS-AAAAAA-K(C19)
**P2-TMZ**	TMZ-PLGSYL-SSS-GKRS-AAAAAA-K(C19)
**P2-BrP**	BrP- K[TPP]-PLGSYL-SSS-GKRS-AAAAAA-K(C19)
**P2-TPP+-FITC**	FITC- K[TPP]-PLGSYL-SSS-GKRS-AAAAAA-K(C19)

Regarding the drugs BrP and TMZ, both were covalently linked to the MMP-9 cleavage sequence “PLGSYL”, which is bound to P2 through a linker of three serines. Before the drug attachment, the peptide sequence was elongated using the SPPS protocol including coupling reactions and Fmoc removals under ultrasound irradiations, and also the lipid tail C19 was added as described above.

The attachment of TMZ on peptide P2-TMZ was performed overnight after the Fmoc removal from the N-terminus and the coupling between 3-methyl-4-oxo-3,4-dihydroimidazo­[5,1-*d*]^1–3,5^-tetrazine-8-carboxylic acid (temozolomide acid) and free amine was carried out in DMF with 1-ethyl-3-(3-(dimethylamino)­propyl)­carbodiimide (EDC, 2 equiv) and 4-dimethylaminopyridine (DMAP, 0.1 equiv).^10^ The attachment was confirmed by performing HPLC and ESI-MS analysis.

Instead, BrP conjugation involved several steps. The first step consisted of the incorporation of mitochondrial moiety TPP^+^ in the N-terminus on the lysine side chain. In this case, Fmoc-Lys­(Mtt)-OH was added in the N-terminus after the C19 incorporation, and the Mtt group was deprotected as described above. Then, (2-carboxyethyl)­triphenylphosphonium bromide (3 equiv, TPP^+^) was conjugated on free amine using HOBt (3 equiv), HBTU (3 equiv), and DIPEA (6 equiv), in NMP overnight. After monitoring the TPP^+^ conjugation by HPLC and ESI-MS analysis, we removed the Fmoc group from the N-terminus and the BrP preactivated using EDC (3 equiv) and NHS (4 equiv) in NMP for 30 min was added on the resin for 6 h at rt.^51^ The BrP coupling was ascertained by a colorimetric Kaiser test and ESI-MS analysis.

#### 4.2.3 Labeled Peptide Synthesis

For the uptake experiments useful to evaluate the nanofiber internalization, we synthesized the peptides P2-f and P2-TPP^+^-FITC labeled with 5(6)-carboxy-fluorescein (FITC).

Regarding the synthesis of P2-f, before the FITC attachment, we attached the linker Fmoc-PEG2-OH to the peptide P2 by performing two coupling reactions: (i) Fmoc-PEG2-OH (2 equiv), DIC (2equiv), Oxyma (2 equiv), DMF, 2 h; (ii) Fmoc-PEG2-OH (2 equiv), HATU (2equiv), DIPEA (4 equiv), DMF, 2 h. Then, Fmoc was removed as described above, and FITC was bound treating with COMU (2 equiv), Oxyma (2 equiv), and DIPEA (4 equiv) under stirring for 25 min.^10^ Two couplings were performed. The FITC labeling was determined by using HPLC and ESI-MS analyses. This protocol for FITC conjugation was also used for labeling the peptide P2-TPP^+^-FITC. Specifically, after the coupling of TPP^+^ on lysine side chain as described above, we removed the Fmoc group and FITC was added to the resin with COMU (2 equiv), Oxyma (2 equiv), and DIPEA (4 equiv).

#### 4.2.4 Peptide Purification

Following full synthesis, all peptides and protecting groups were separated from the resin by subjecting them to a 3 h stirring treatment with the acid mixture TFA:TIS:H_2_O (95:2.5:2.5, v:v:v). After filtering the resin, cold diethyl ether (Et_2_O) was used to precipitate each peptide, and the mixture was centrifuged twice for 15 min at 6000 rpm. Crude peptides were dissolved in 1,1,1,3,3,3-hexafluoro-2-propanol (HFIP) (20%) and H_2_O (0.1% TFA) and purified by preparative high-performance liquid chromatography (HPLC) on a Phenomenex Kinetex C18 column (5 μm, 100 Å, 150 × 21.2 mm) using linear gradients of MeCN (0.1% TFA) in water (0.1% TFA), from 10 to 90% over 35 min, with a flow rate of 15 mL/min and UV detection at 220 nm. Peptide purity was assessed by analytical HPLC (Jasco LC-4000) using a Phenomenex Jupiter Proteo column (90 Å, 150 × 4.6 mm), and peptide identity was confirmed by ESI-MS analysis (Figures S5–S16).

### 4.3 Nanofiber Preparation and Characterization

#### 4.3.1 Peptide Assembly

The peptide assembly in solution was investigated by using fluorescence-based assays and Nile Red and thioflavin T as fluorescent dyes. First, the critical aggregation concentrations of the nanofibers NF-BrP (P1+P2+P2-t+P3+P2-BrP, 1:0.64:0.06:0.2:0.1) and NF-TMZ-BrP (P1+P2+P2-t+P3+P2-BrP+P2-TMZ, 1:0.54:0.06:0.2:0.1:0.1) were evaluated using the dye Nile Red (NR) since it is able to incorporate in hydrophobic environments such as the inner core of peptide aggregates, producing a blue shift and an increase in the fluorescence intensity.^52^ For each PA, we prepared concentrated stock solutions in 1,1,1,3,3,3-esafluoro-2-propanolo (HFIP), and the NR assay was performed coassembling the PAs with each other at the specific ratio and formulating the nanofiber at different concentrations of 0.5, 0.8 1, 3, 5, 7, 10, 15, 20, 25, 30, 50, 100, 150, and 200 μM. The organic solvent was eliminated under a nitrogen stream, the water was added, and each formulation was lyophilized. For the CAC calculation, each nanofiber was hydrated with NR solution (500 nM) for 1 h. All NR fluorescence spectra were obtained using a Cary Eclipse fluorescence spectrometer (Agilent, Milan, Italy), with excitation of 550 nm and emission spectra collected between 570 and 700 nm. CAC values were determined reporting the wavelength of the maximum fluorescence intensity against peptide concentration and fitting the resulting curve exploiting the sigmoidal Boltzmann equation:

$$y=\frac{A_{1}+A_{2}}{1+e^{\left(x-x_{0}/\Delta x\right)}}+A_{2}$$

In the equation, *A*_1_ and *A*_2_ indicate the upper and lower limits of the sigmoid, respectively, whereas *x*_0_ and Δ*x* are the inflection point and steepness of the sigmoid function, respectively.

In addition, the nanofiber assembly in solution was also monitored by exploiting thioflavin T (ThT). ThT is a benzothiazole tool commonly used to measure the aggregation and specifically the amyloid fibril formation.^53^ When ThT binds to aggregates, it exhibits enhanced fluorescence at 482 nm. We performed the ThT experiment preparing each nanofiber at 100 μM. The nanofiber was hydrated in water, and after 1 h, ThT at 25 μM was added to the nanofiber. Each sample was recorded exciting the ThT at 450 nm (slit width: 5 nm), and fluorescence emission was recorded at 482 nm (slit width: 10 nm).

#### 4.3.2 Zeta Potential Measurements

The zeta potential of each nanofiber sample was measured by using a Zetasizer Nano-ZS (Malvern Instruments, Worcestershire, UK). Nanofibers were prepared at a concentration of 50 μM as previously described above. Measurements were performed at 25 °C using a 4 mW He–Ne laser operating at 633 nm, with a fixed scattering angle of 173°.

#### 4.3.3 Nanofiber Characterization

Hydrophobic chain organization within the nanofiber inner core was investigated by electron paramagnetic resonance (EPR) spectroscopy using two spin-labeled fatty acids, 5-DSA and 16-DSA, as molecular spin probes.^10^ The spin probes were incorporated into the nanofibers by adding an appropriate aliquot of a 1 mg mL^–1^ ethanolic spin-probe solution to the peptide mixture in HFIP prior to drying and rehydration. Final concentrations of nanofiber and spin probe were 100 μM and 1 μM, respectively. A 20 μL aliquot of each suspension was transferred into glass capillaries, flame-sealed, and inserted into standard 4 mm quartz EPR tubes containing light silicone oil to ensure thermal stability. EPR spectra were acquired at 25 °C using a 9 GHz Bruker Elexsys E500 spectrometer (Bruker, Rheinstetten, Germany) equipped with a superhigh sensitivity probehead. Temperature control was achieved via a quartz dewar flushed with thermostated nitrogen gas. The instrument settings were as follows: sweep width, 90 G; modulation frequency, 100 kHz; modulation amplitude, 1.0 G; time constant, 20.48 ms; conversion time, 20.48 ms; incident power, 5.0 mW. To enhance the signal-to-noise ratio, 128 scans were accumulated. Spectral analysis focused on determining the outer hyperfine splitting (2Amax), defined as the difference between the low-field maximum and high-field minimum, which provides a quantitative estimate of the mobility and ordering of the spin-labeled hydrophobic tail segment.^54^

#### 4.3.4 Circular Dichroism Spectroscopy

The analysis of the peptide secondary structure in the nanofibers NF-BrP and NF- TMZ-BrP was performed by circular dichroism (CD) spectroscopy under different conditions. Specifically, we prepared the nanofiber at the concentration of 50 μM and evaluated its stability to the dilution effect at the concentrations of 40, 30, and 20 μM. In addition, we also monitored the nanofiber stability (25 μM) under pH environments (pH 3 and 10) and the ionic strength, changing the concentration of sodium chloride (NaCl) from 1 to 10 mM. CD spectra were recorded from 195 to 260 nm at room temperature by exploiting a Jasco J-810 spectropolarimeter equipped with a 1.0 cm quartz cuvette. Each spectrum represents the average of three scans and is reported in terms of the molar ellipticity.

#### 4.3.5 Trp Quenching by Acrylamide

The presence and exposure of the cell-penetrating peptide gH625 on the nanofiber surface was monitored by performing the tryptophan (Trp) quenching by the quencher acrylamide. Each nanofiber formulation, NF without drugs, NF-BrP, and NF-TMZ-BrP, was prepared at 200 μM with the peptide P3 carrying gH625 at the concentration of 10 μM. After 1 h of hydration in water, the nanofiber was quenched with acrylamide at concentrations from 0.02 to 0.4 M. Each Trp spectrum was recorded setting the fluorescence excitation at 295 nm. The accessibility of Trp of the peptide gH625 was determined by calculating the Stern–Volmer quenching constant and analyzing the data with the following Stern–Volmer equation: *F*_0_/*F* = 1 + *K*_sv_[Q], where *F*_0_ and *F* indicate the fluorescence intensities in the absence and the presence of the quencher (Q), respectively.^55^

#### 4.3.6 Lipid Mixing Assay

The membrane fusion activity of the peptide gH625 was evaluated using large unilamellar vesicles (LUVs) composed of phosphatidylcholine (PC) and cholesterol (Chol) in a 1:1 molar ratio to mimic the composition of eukaryotic membranes. We prepared LUVs labeled with rhodamine (Rho) and nitrobenzoxadiazole (NBD) as fluorophores to perform the resonance energy transfer assay. Specifically, we prepared LUVs made of PC:Chol 1:1 at the concentration of 0.16 mM and LUVs made of PC:Chol with 0.6% mol of NBD-PE and 0.6% mol of Rho-PE at the concentration of 0.04 mM.^56^ For the evaluation of fusogenic activity, unlabeled and labeled LUVs were mixed at a 4:1 ratio to obtain a final lipid concentration of 0.1 mM. The nanofibers (NF, NF-BrP, NF-TMZ-BrP) were prepared at the concentration of 400 μM in water with the peptide P3 at the concentration of 40 μM. After 1 h of hydration in water, each nanofiber was added to LUVs at the concentrations of 5, 10, 15, 20, 30, and 50 μM, corresponding to concentrations of the exposed peptide gH625 of 0.5, 1, 2, 3, and 5 μM. The lipid mixing was evaluated by recording the NBD emission at 530 nm and Rho emission at 590 nm followed with the NBD excitation wavelength set at 465 nm.^57^ The percentage of fusion after each nanofiber addition was calculated as a function of 100% value, corresponding to complete mixing of lipids upon the addition of Triton X-100 (0.05% v/v).^57^ The percentage fusion was calculated as

$$\%\;\mathrm{Fusion}=\frac{[F530/F590{]}_{peptide}-[F530/F590{]}_{blank}}{[F530/F590{]}_{triton}-[F530/F590{]}_{blank}}\times 100$$

where *F*530 and *F*590 are the fluorescence intensities at 530 and 590 nm calculated in the absence and in the presence of the peptide and Triton X-100.

#### 4.3.7 Scanning Electron Microscopy Analysis

The size and morphology of nanofiber formulations were analyzed by scanning electron microscopy (SEM). Nanofibers NF-BrP and NF-TMZ-BrP were formulated at a concentration of 50 μM, and 5 μL of each formulation was deposited onto a cleaned silicon wafer and air-dried at room temperature. Images were acquired using a dual beam FIB-SEM Aquilos 2 instrument by ThermoFisher Scientific (Milan, Italy). The acquisition parameters were as follows: current 0.2 nA, voltage 7.5 kV, working distance 2.6 mm, field of view 4.14 μm, stage tilt 0, and magnification 50000×.

### 4.4 Functional Biological Studies

#### 4.4.1 Nanofiber Preparation for Biological Studies

All nanofiber formulations (Table 3) used for the biological studies were prepared at a final concentration of 100 μM. Stock solutions of PAs were initially dissolved in HFIP and coassembled at the specific ratios required for nanofiber formation. The HFIP was eliminated under a nitrogen stream, and each formulation was subsequently lyophilized overnight in the presence of 1 mL of water. Nanofiber assembly was initiated by rehydration in either water or cell culture medium, and samples were hydrated for 1 h before the start of each biological experiment. Nanofiber labeled with FITC for uptake experiments was prepared partially replacing P2 with P2-f at the percentage of 6%.

**Table 3:** Nanofiber Formulations

**Formulation**	**Composition**
**NF**	P1+P2+P2-t+P3 (1:0.74:0.06:0.2)
**NF-BrP**	P1+P2+P2-t+P3+P2-BrP (1:0.64:0.06:0.2:0.1)
**NF- TMZ-BrP**	P1+P2+P2-t+P3+P2-BrP+P2-TMZ (1:0.54:0.06:0.2:0.1:0.1)
**NF-TPP+-FITC**	P1+P2+P2-t+P3+P2-TPP+-FITC (1:0.64:0.06:0.2:0.1)
**FITC-NF-BrP**	P1+P2+P2-t+P2-f+P3+P2-BrP (1:0.52:0.06:0.12:0.2:0.1)
**FITC-NF-BrP-TMZ**	P1+P2+P2-t+P2-f+P3+P2-BrP+P2-TMZ (1:0.42:0.06:0.12:0.2:0.1:0.1)

#### 4.4.2 Cell Uptake Evaluation

To evaluate NF cell uptake, fluorescent NFs (labeled with FITC) were added to U-87 MG cells grown on 4-well chamber slides (Sarstedt) at different concentrations and different times. Cells were seeded in chamber slides at 3 × 10^4^ cells/chamber density and treated with complete fiber (FITC-NF-BrP and FITC-NF-TMZ-BrP) for 30, 60, and 90 min. After different uptake tests, the final concentration of BrP 5 μM and TMZ 5 μM was established. During the last 30 min of the 90 min treatment (chosen as the optimal time), Mito Tracker Deep Red dye (ThermoFisher Scientific), a mitochondrion-selective fluorescent dye that accumulates in mitochondrial membranes, was added to the culture medium at a final concentration of 300 nM. Cells were fixed with 4% paraformaldehyde for 30 min, washed with 0.1 M PBS, and counterstained with Hoechst (10 μg/mL). Following mounting with IBIDI aqueous mounting medium, samples were imaged using an Axioskop microscope (Carl Zeiss, Germany) and image acquisition was performed with ZEN 3.8 software (Zeiss). Image analysis was carried out using Zen (Zeiss) and Fiji software to evaluate nanofiber uptake by cells and to assess changes in the cell morphology following treatment.

#### 4.4.3 2D Cell Treatments and Viability Assay

Cells were seeded in 48-well plates at approximately 3 × 10^4^ cells per well in DMEM culture medium supplemented with 10% FBS. U-87 MG cell lines from human brain were initially cultured in 25 cm^2^ flasks, using DMEM supplemented with FBS 10%, P/S 2%, and L/GLUT 2 mM, in a humidified incubator (37 °C/5% CO_2_). After 24 h, once cells adhered, they were treated in parallel with NF-BrP (BrP, 5 μM), NF-BrP-TMZ (BrP and TMZ, 5 μM), and BrP at concentrations of 30, 50, 100, and 150 μM, for 24, 48, and 72 h. Untreated cells served as the controls. All experiments were performed under standard cell culture conditions (95% relative humidity, 5% CO_2_, 37 °C). At the end of each incubation time, cell viability was assessed using the PrestoBlue assay (ThermoFisher Scientific), according to the manufacturer’s protocol. Cells were incubated with the reagent for 1 h, and absorbance was obtained at 570 nm with 600 nm as the reference wavelength with a BioTek Synergy HT microplate reader. Results are expressed as the percentage of viable cells relative to the untreated controls.

An assessment of HBMEC cell survival was performed to determine whether NF-BrP (5 μM) and NF-TMZ-BrP (BrP and TMZ, 5 μM) impact on brain endothelial cells’ health expressing EGFRs.^58^ Cells were plated in a 96-well plate (15000 cells/well) in their cell growth medium. Once cells adhered, they were treated with NF-BrP and NF-TMZ-BrP every 24 h for 3 days. The treatment concentration was determined based on preliminary experiments conducted on 2D U87 cell lines. HBMEC control cells were grown in their appropriate medium culture without treatment. Cell viability was measured using the PrestoBlue assay under the same conditions outlined above for 2D U87. To evaluate the release of BrP and TMZ on HBMEC, cells were treated with MMP-9 preactivated by APMA 100 μM and Tris-HCl 50 mM (pH 7.2) at 37 °C for 3 h.^59^ NF-BrP (BrP, 5 μM) and NF-TMZ-BrP (TMZ and BrP, 5 μM) were hydrated in this buffer solution: 50 mM HEPES, 200 mM NaCl, 10 mM CaCl_2_, and 1 mM ZnCl_2_, at pH 7. Subsequently, activated MMP-9 was added to NF-BrP and NF-TMZ-BrP at a final concentration of 40 nM and added to the medium cells for 72 h. Viability effects were evaluated using the PrestoBlue assay as reported above.

#### 4.4.4 A Dynamic in Vitro 3D BBB Model under Flow Conditions

The entire setup of the in vitro 3D BBB fluid dynamic model was carried out as reported elsewhere.^10,37^ Briefly, in the upper chamber of the Livebox 2 (LB2) bioreactor a coculture of HBMEC and hPC-PL was seeded on a porous membrane;^60^ 3D U87 cells were bioprinted using a droplet-based approach.^61^ A cell suspension (1 × 10^5^/mL) was prepared in complete DMEM medium and loaded into a sterile syringe cartridge. Droplets were extruded through a 27-gauge nozzle using a pneumatic pressure of 9 kPa and a printing speed of 1 mm/s, using a BioX (CELLINK, Sweden) bioprinter. Bioprinting was performed directly into ultralow-attachment 6-well plates to prevent cell adhesion and promote the self-assembly of cells into 3D spheroidal structures. Constructs were incubated under standard culture conditions (37 °C and 5% CO_2_). After 24 h, 3D cellular aggregates were carefully transferred into the lower chamber of LB2 for 24 h under a flow rate of 120 μL/min ensuring no shear stress on the cells.

To assess the passage of FITC-NF-BrP (BrP, 5 μM) or FITC-NF-TMZ-BrP (TMZ and BrP, 5 μM) across the BBB model, each formulation was administered into the upper chamber at the membrane interface. A fluorescence-based spectrofluorimetric assay was conducted by collecting medium samples (100 μL) at specific time points (30, 90, and 180 min) from both the upper and lower chamber outlet tubes and transferring them into a 96-well plate. Fluorescence intensity was measured using a Bio-Tek Synergy HT microplate reader (USA) (*l*_ex_: 491 nm; *l*_em_: 516 nm).

#### 4.4.5 Cell Viability Assay on the 3D Dynamic in Vitro BBB Model

To investigate the impact of NF-BrP and NF-TMZ-BrP on 3D GBM cells cultured under dynamic flow conditions, in the LBs2, treatment with NF-BrP (5 μM) or NF-TMZ-BrP (BrP and TMZ, 5 μM) was administered into the upper chamber once every 24 h over a 3-day period. The viability of 3D U87 following treatment was assessed by using the PrestoBlue cell viability reagent. Briefly, after each treatment, spheroids were retrieved from the lower chamber and transferred to a 48-well plate. PrestoBlue reagent, diluted 1:10 in culture medium, was added to each well and incubated for 180 min. Absorbance was then measured using a Bio-Tek Synergy HT microplate reader (570–600 nm). As a control, an identical LB2 setup was used without treatment.

#### 4.4.6 Mitochondria Isolation from Rat Brain and Mitochondria Respiration

Male Wistar rats (275–300 g) were obtained from Envigo RMS Srl (Udine, Italy). Animals were housed in a temperature-controlled environment (22 °C) under a 12:12 h light–dark cycle, with food and water available ad libitum. All procedures were conducted in strict accordance with European guidelines for the care and use of laboratory animals. Every attempt was made to reduce the suffering and discomfort experienced by the animals. Experimental protocols were approved by the Committee on the Ethics of Animal Experiments of the University of Naples Federico II (Italy) and the Italian Minister of Health (protocol number 776/2021-Pr). Rats were anesthetized by ip injection of tiopental (40 mg/100 body weight) and euthanized by decapitation. Brains were immediately excised, weighed, and processed for mitochondrial isolation, following the method described by Sumbalova et al.^62^ Briefly, brain tissues were immersed in ice-cold isolation buffer (330 mM sucrose, 10 mM Tris-HCl, 1 mM EDTA, and 2.5 g/L fatty acid-free BSA, pH 7.4) and homogenized using a Potter-Elvehjem homogenizer. The homogenate was centrifuged at 1000*g* for 10 min at 4 °C, and the obtained supernatant was subsequently centrifuged at 62000*g* for 10 min at 4 °C to obtain a mitochondrial-enriched pellet. Mitochondrial pellets were washed twice in the above buffer without BSA and resuspended in a minimal volume of the isolation medium. Mitochondrial respiration rate was measured polarographically using a Clark-type electrode (Clark-type electrode, Oxygraph system, Hansatech Instruments Ltd., UK). Mitochondria (100 μg proteins) were incubated in 0.5 mL of respiration buffer (0.80 mM KCl, 50 mM HEPES (pH 7.0), 1 mM EGTA, 5 mM K_2_HPO_4_, 0.1% BSA (wt/vol)) at 37 °C for 8 min in the presence of vehicle, BrP, NF, or NF-Br, after which the respiration was initiated by adding pyruvate (5 mM) + malate (2.5 mM) or succinate (5 mM) (in the presence of 4 μM rotenone an inhibitor of complex I). After 8 min, ADP (300 μM) was added to the incubation medium.

#### 4.4.7 EPR Analysis of ROS Production upon Mitochondria Respiration

Investigation of the redox status in mitochondria was performed using the Mito-TEMPO paramagnetic spin probe, which is a mitochondria-penetrating nitroxide radical that allows evaluation of the level of ROS (mainly superoxides) produced during respiration.^63^ The same protocol as described in the previous subsection was followed with the addition of Mito-TEMPO (0.1 mM) during the preincubation with BrP, NF, or NF-Br. In this case succinate was used as a respiratory substrate in the absence of rotenone in order to allow the formation of ROS via the electron reverse from complex II to complex I. At predetermined time intervals, 20 μL of each sample was loaded into glass capillaries and flame-sealed. To guarantee thermal stability, the sealed capillaries were subsequently placed inside a standard 4 mm quartz EPR-sample tube that had been lightly lubricated with silicone oil. As described in a previous subsection, a 9 GHz Bruker Elexsys E500 spectrometer was used to record the EPR spectra. During acquisition, the sample temperature was maintained at 37 °C by directing thermostated nitrogen gas through a quartz dewar. Mito-TEMPO EPR signals were analyzed by double integration using the Bruker software package.

#### 4.4.8 Annexin V-FITC/Propidium Iodide Assay

To assess apoptosis and necrosis following 72 h treatment in the 3D dynamic in vitro BBB model, a dual staining assay using Annexin V-FITC and propidium iodide (PI) was performed on 3D U87 cells, according to the manufacturer’s instructions. Following incubation with the dual staining solution, nuclear staining was performed using 4′,6-diamidino-2-phenylindole (DAPI). Image acquisition was carried out using a JuLi Stage real-time cell history recorder microscope (NanoEntek, Singapore) equipped with a 10× objective. Fluorescence images were captured by using the following filter sets: GFP channel, Annexin V-FITC (*l*_ex_: 395 nm, *l*_em_: 475 nm); RFP channel, PI (*l*_ex_: 550 nm, *l*_em_: 650 nm); DAPI channel, nuclei (*l*_ex_: 358 nm, *l*_em_: 461 nm). Captured images were processed using Fiji software to adjust brightness and contrast and were analyzed to evaluate cellular morphology post-treatment. Each experimental condition was repeated in three independent assays.

#### 4.4.9 Morphological Analysis of 3D U-87 Cells

Spheroid images were acquired by a JuliStage cell history recorder microscope through 72 h of treatment. At different time points (24, 48, and 72 h), whole well images were acquired and used to evaluate shape descriptors by ImageJ software. Area, Feret’s diameter, defined as the longest (Feret max) and shortest (Feret min) distance along the selection border between any two points, and roundness, defined as

$$Roundness=4\frac{\left[Area\right]}{\pi {\left[Major\;axis\right]}^{2}}$$

of the spheroids were estimated for each experimental class.

#### 4.4.10 Statistical Analyses

The results are shown as means ± SEM, and each experiment was run in triplicate. For both 2D and 3D cell experiments, statistical analysis was conducted using one-way analysis of variance (ANOVA) followed by Dunnett’s post-test. For morphological analysis, statistical significance between groups was assessed by a one-way ANOVA, with Bonferroni’s multiple comparisons post-test. Data were considered statistically significant at *p < 0.05, **p < 0.01, and ****p < 0.0001. To compare treated groups with corresponding controls, the two-tailed Mann–Whitney test was applied. As for the Annexin/PI test, statistical significance was determined using the Kruskal–Wallis nonparametric test with Dunn’s comparison as post-test. Significance thresholds were set at **p < 0.01, ***p < 0.001, and ****p < 0.0001. All graphs were generated using GraphPad Prism software.

## Supplementary Material



## Notes

Microscopy images of NF-BrP-FITC (Figure S1); immunolocalization of MMP-9 in U-87 cells (Figure S2); BrP cytotoxicity after treatment for 24 h (Figure S3); dose–response of BrP alone on mitochondrial function (Figure S4); HPLC chromatograms and ESI-MS spectra of peptides (Figures S5–S16) (PDF)

## ABBREVIATIONS

ADP: adenosine diphosphate
ATP: adenosine triphosphate
BBB: blood–brain barrier
BrP: 1,3-bromopyruvate
CD: circular dichroism
GBM: glioblastoma
HBMEC: healthy brain endothelial cells
hPC-PL: human pericytes
LUVs: large unilamellar vesicles
MMP-9: matrix metalloproteinase-9
NF: nanofiber
PAs: peptide amphiphiles
ROS: reactive oxygen species
ThT: thioflavin
TMZ: temozolomide
TEMPO: tetramethylpiperidinyl-oxyl) radical
TPP^+^: triphenylphosphonium.
