# Supplementary material for: Synergistic Cancer Metabolic Therapy via Co-Delivery of 3‑Bromopyruvate and Temozolomide with a Supramolecular Shuttle
Source: ACS Appl Mater Interfaces. 2025 Oct 22;17(44):60342–60. doi: 10.1021/acsami.5c17607 (PMC12598698; doi:10.1021/acsami.5c17607)
Supplement: Supplementary file 1 [file am5c17607_si_001.pdf]

Supporting information

Synergistic Cancer Metabolic Therapy via Co-Delivery of 3-Bromopyruvate and Temozolomide with a Supramolecular Shuttle

Rosa Bellavita,[1] Marina Prisco,[2] Sara Palladino,[1] Teresa Barra,[1,2] Federica Donadio,[3] Emanuela Esposito,[3] Rodolfo Esposito,[4] Giuliana Panico,[2] Jessica Pisano,[2] Paola Venditti,[2] Salvatore Valiante,[2] Annarita Falanga,[5] Gerardino D'Errico,[4] Assunta Lombardi,[2] Stefania Galdiero[1*]

[1]Department of Pharmacy, School of Medicine, University of Naples Federico II, Via Domenico Montesano 49, 80131 Napoli, Italy.

[2]Department of Biology, University of Napoli Federico II, Via Cintia, 80126 Naples, Italy.

[3]Institute of Applied Sciences and Intelligent Systems (ISASI), Naples Cryo Electron Microscopy Laboratory - EYE LAB, National Research Council (CNR), Via Pietro Castellino 111, 80131 Naples, Italy.

[4]Department of Chemical Sciences, University of Napoli Federico II, and CSGI (Unit of Naples), Via Cintia, 80126 Naples, Italy.

[5]Department of Agricultural Sciences, University of Naples Federico II, Via Università 100, Portici, 80055 Portici, Italy

Correspondence: stefania.galdiero@unina.it.

Table of content

1. Microscopy images of NF-BrP-FITC with BrP at 10 μM and 30 μM (Figure S1) 2
2. Immunolocalization of MMP-9 in U-87 cells (Figure S2) 2
4. BrP cytotoxicity after the treatment of 24h (Figure S3) 2
5. Dose–response of BrP alone on mitochondrial function (Figure S4) 3
6. HPLC chromatograms and ESI-MS spectra of peptides (Figure S5-S16) 3-9

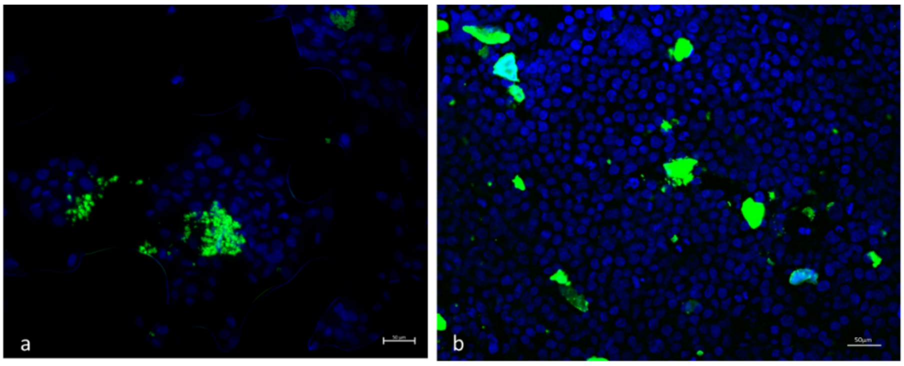

Figure S1. U87 cells treated with NF-BrP-FITC, BrP at 10 μM (a) and 30 μM (b). The nanocarrier does not penetrate cells but forms extracellular aggregates visible as green clusters.

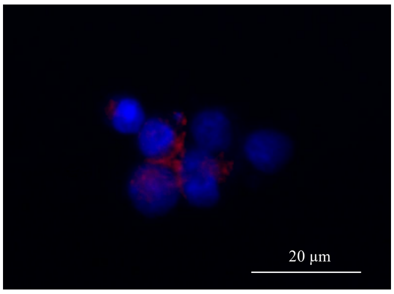

Figure S2. Immunolocalization of MMP-9 in U87 cells. The immunocomplexes are detectable in the cell cytoplasm as red fluorescence.

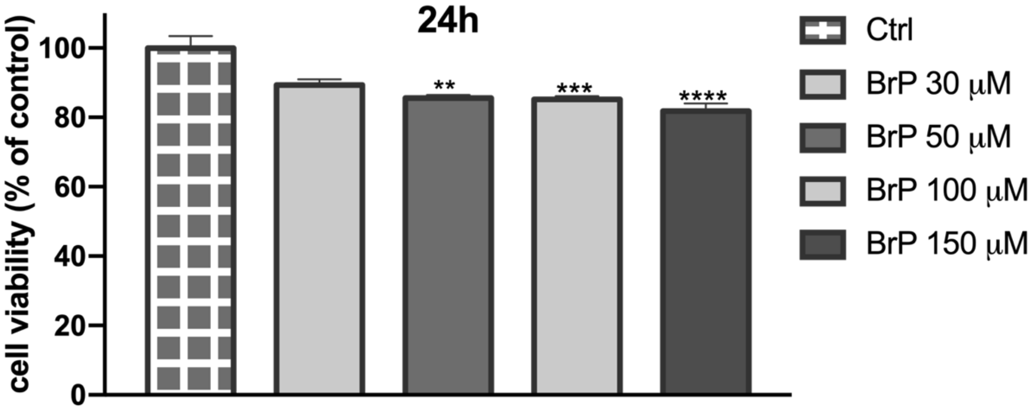

Figure S3. U87 cells were treated with BrP at different concentrations at 24 h. Cell viability is expressed as a percentage of untreated control cells (Ctrl). Values are the means ± SEM of triplicate analysis **p<0.01; ****p < 0.0001.

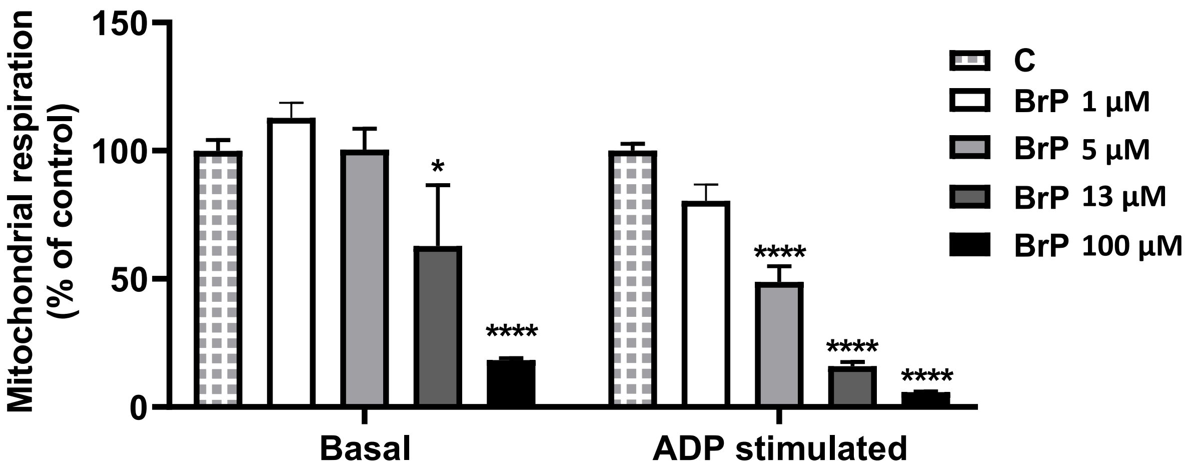

Figure S4. Mitochondrial respiration was detected after the treatment with alone BrP at the concentrations of 1, 5, 13, and 100 µM.

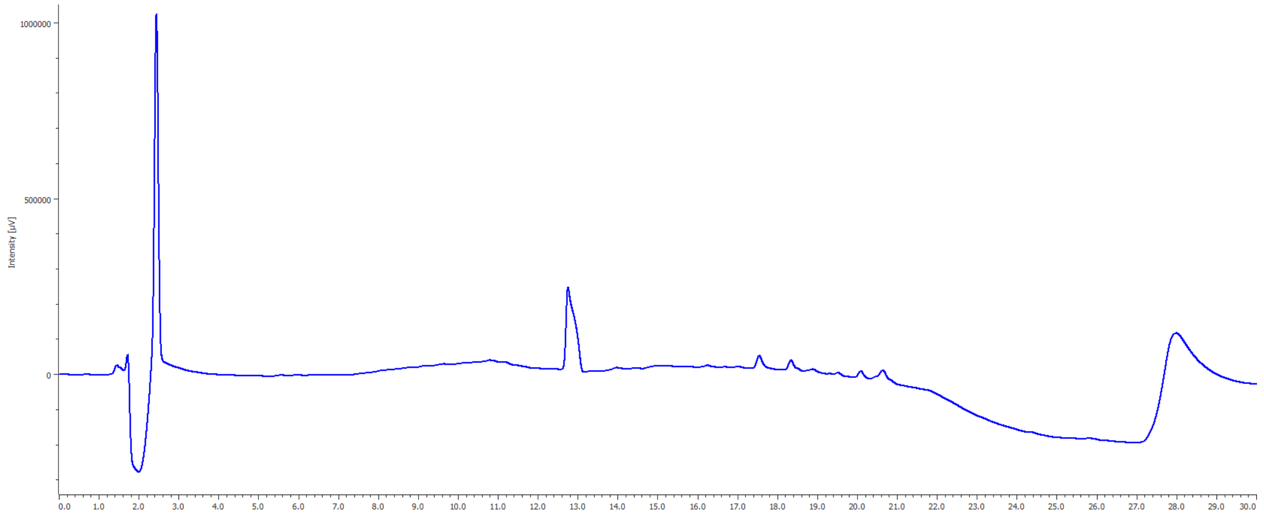

Figure S5. Chromatogram of **peptide P1** obtained by an analytical HPLC (Jasco LC-NetII/ADC) equipped with a Phenomenex Jupiter 4u Proteo column, 90 Å, 150 mm × 4.6 mm [linear gradient 10-90% MeCN (0.1% TFA) in H_2O (0.1% TFA) over 20 min, flow rate of 1 mL/min, and monitored by UV detection at 220 nm.

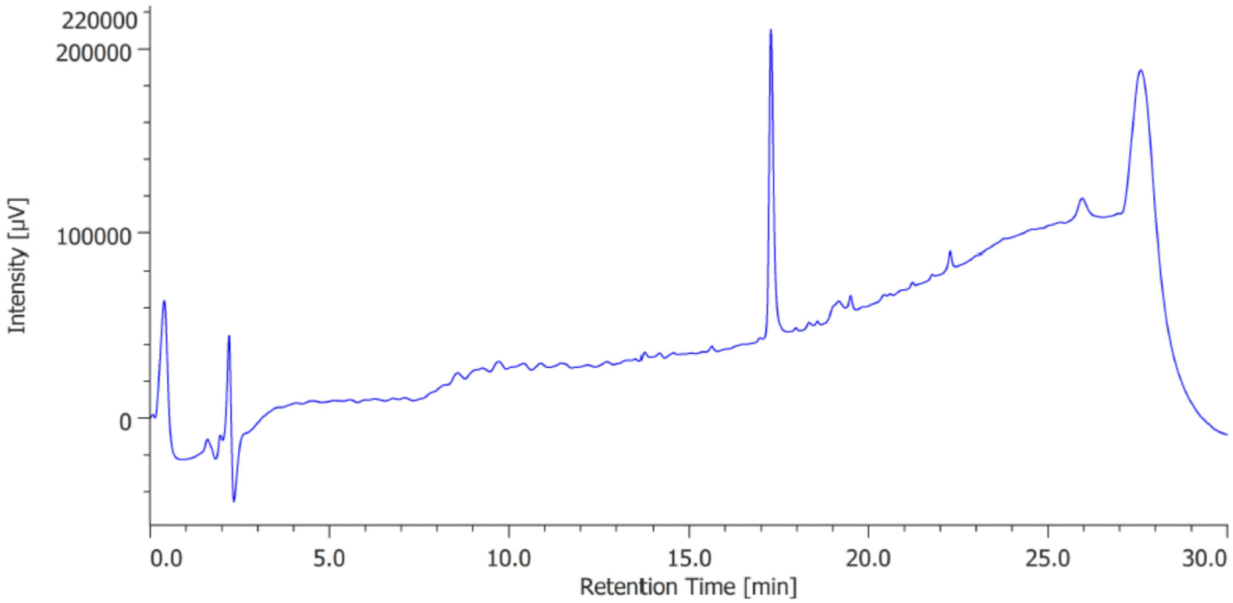

Figure S6. Chromatogram of **peptide P2** obtained by an analytical HPLC (Jasco LC-NetII/ADC) equipped with a Phenomenex Jupiter 4u (C12) Proteo column, 90 Å, 150 mm × 4.6 mm [linear gradient 10-90% MeCN (0.1% TFA) in H_2O (0.1% TFA) over 20 min, flow rate of 1 mL/min, and monitored by UV detection at 220 nm.

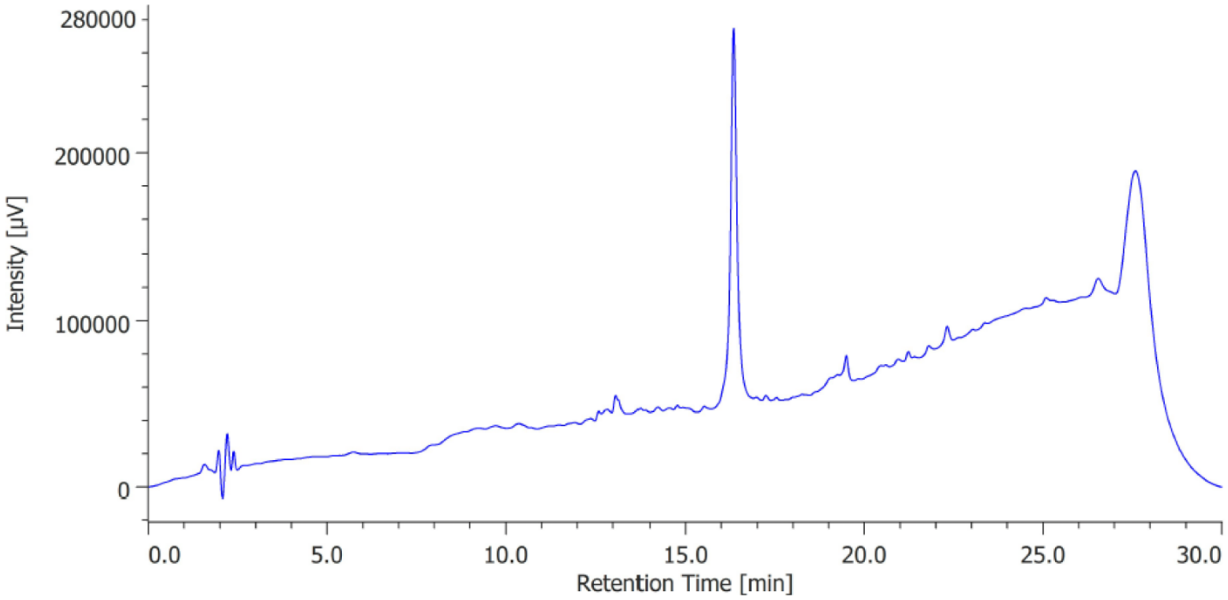

Figure S7. Chromatogram of **peptide P3** obtained by an analytical HPLC (Jasco LC-NetII/ADC) equipped with a Phenomenex Jupiter 4u Proteo column, 90 Å, 150 mm × 4.6 mm [linear gradient 10-

90% MeCN (0.1% TFA) in H_2O (0.1% TFA) over 20 min, flow rate of 1 mL/min, and monitored by UV detection at 220 nm.

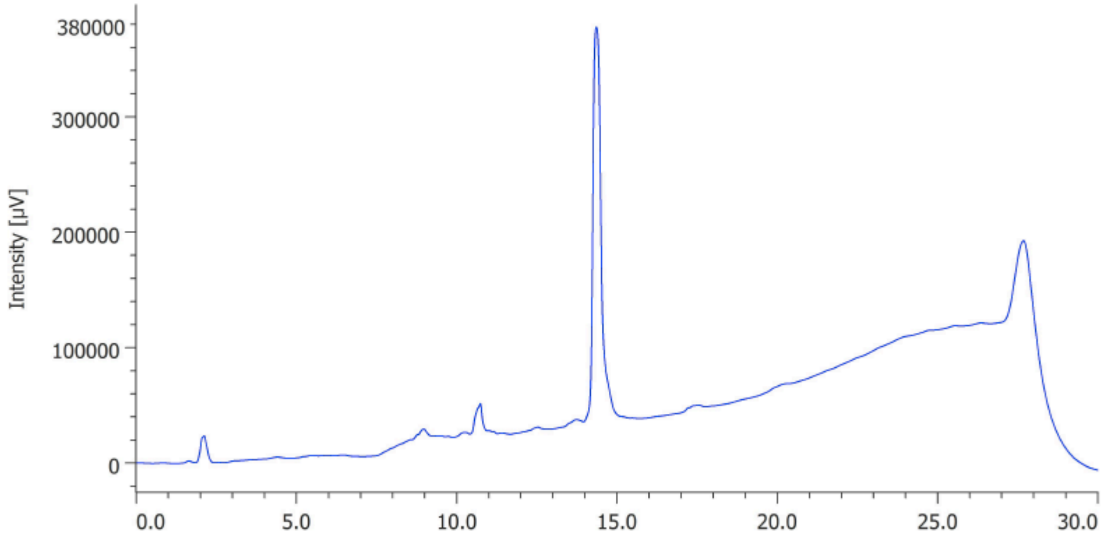

Figure S8. Chromatogram of **peptide P2-t** obtained by an analytical HPLC (Jasco LC-NetII/ADC) equipped with a Phenomenex Jupiter 4u Proteo column, 90 Å, 150 mm × 4.6 mm [linear gradient 10-90% MeCN (0.1% TFA) in H_2O (0.1% TFA) over 20 min, flow rate of 1 mL/min, and monitored by UV detection at 220 nm.

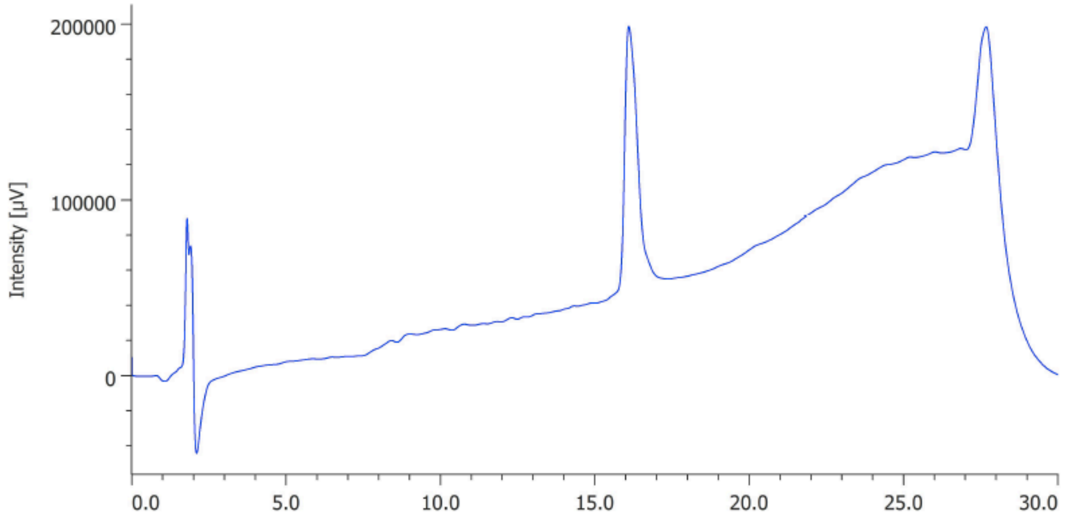

Figure S9. Chromatogram of **peptide P2-TMZ** obtained by an analytical HPLC (Jasco LC-NetII/ADC) equipped with a Phenomenex Jupiter 4u Proteo column, 90 Å, 150 mm × 4.6 mm [linear

gradient 10-90% MeCN (0.1% TFA) in H_2O (0.1% TFA) over 20 min, flow rate of 1 mL/min, and monitored by UV detection at 220 nm.

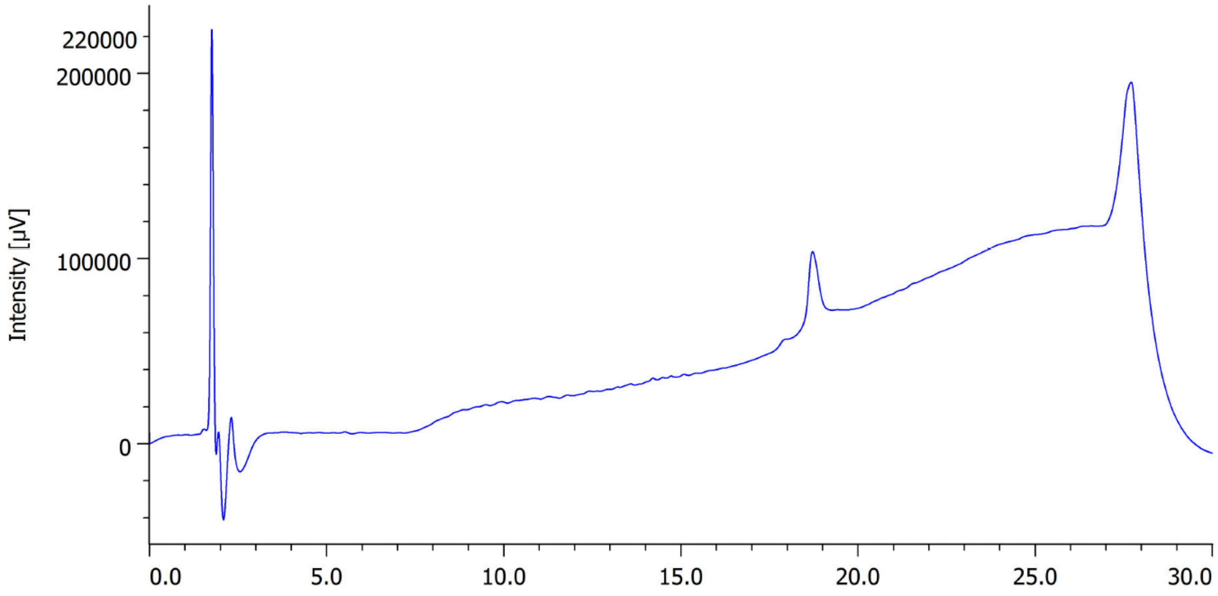

Figure S10. Chromatogram of **peptide P2-BrP** obtained by an analytical HPLC (Jasco LC-NetII/ADC) equipped with a Phenomenex Jupiter 4u Proteo column, 90 Å, 150 mm × 4.6 mm [linear gradient 10-90% MeCN (0.1% TFA) in H_2O (0.1% TFA) over 20 min, flow rate of 1 mL/min, and monitored by UV detection at 220 nm.

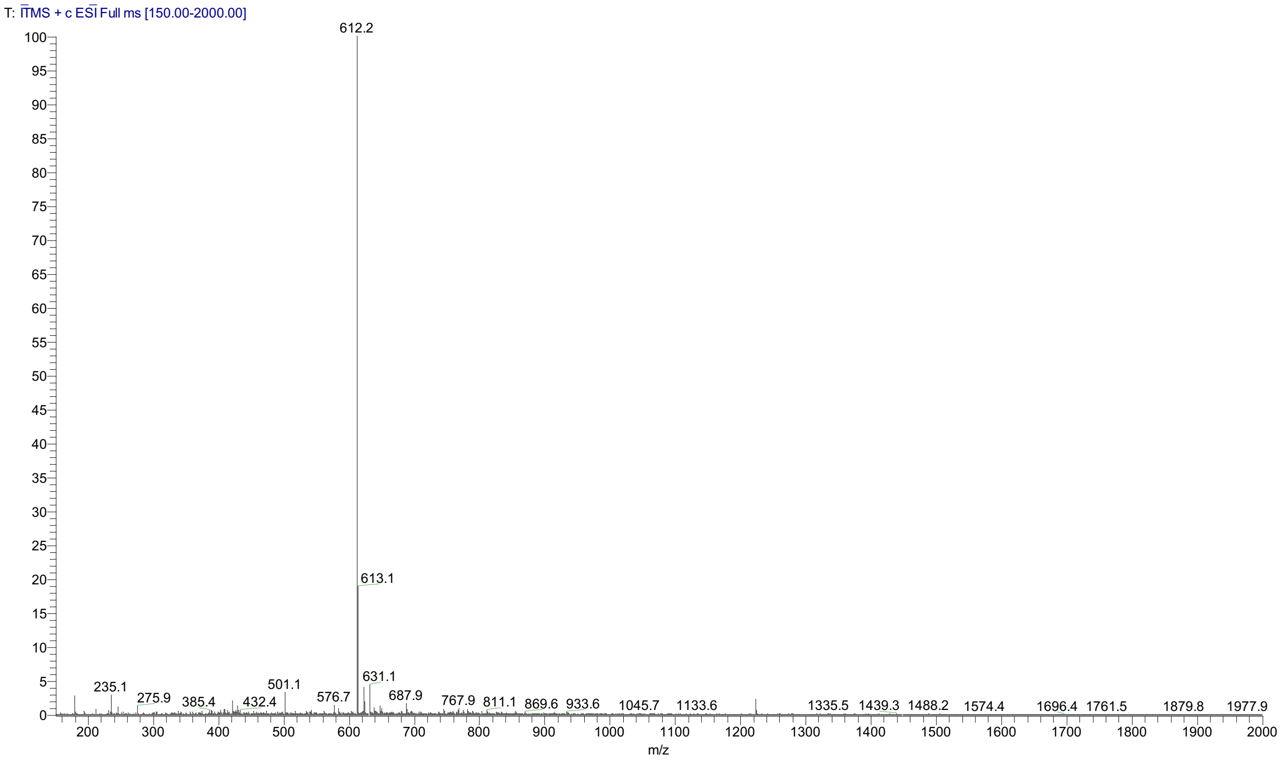

Figure S11. ESI-MS of peptide **P1**. Calculated mass: $[M-2H]^+/2$= 612.3. Found mass: $[M-2H]^+/2$= 612.1.

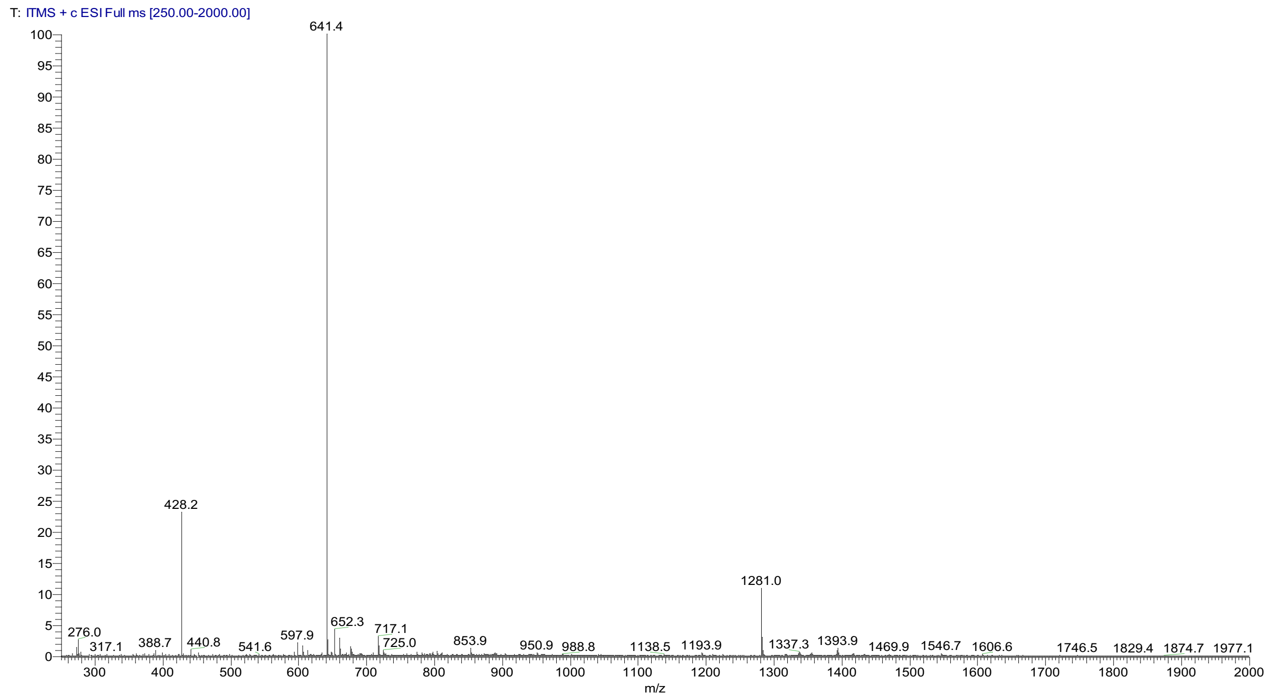

Figure S12. ESI-MS of peptide **P2**. Calculated mass: $[M+2H]^{+}/2$= 641.3. Found mass: $[M+2H]^{+}/2$= 641.4.

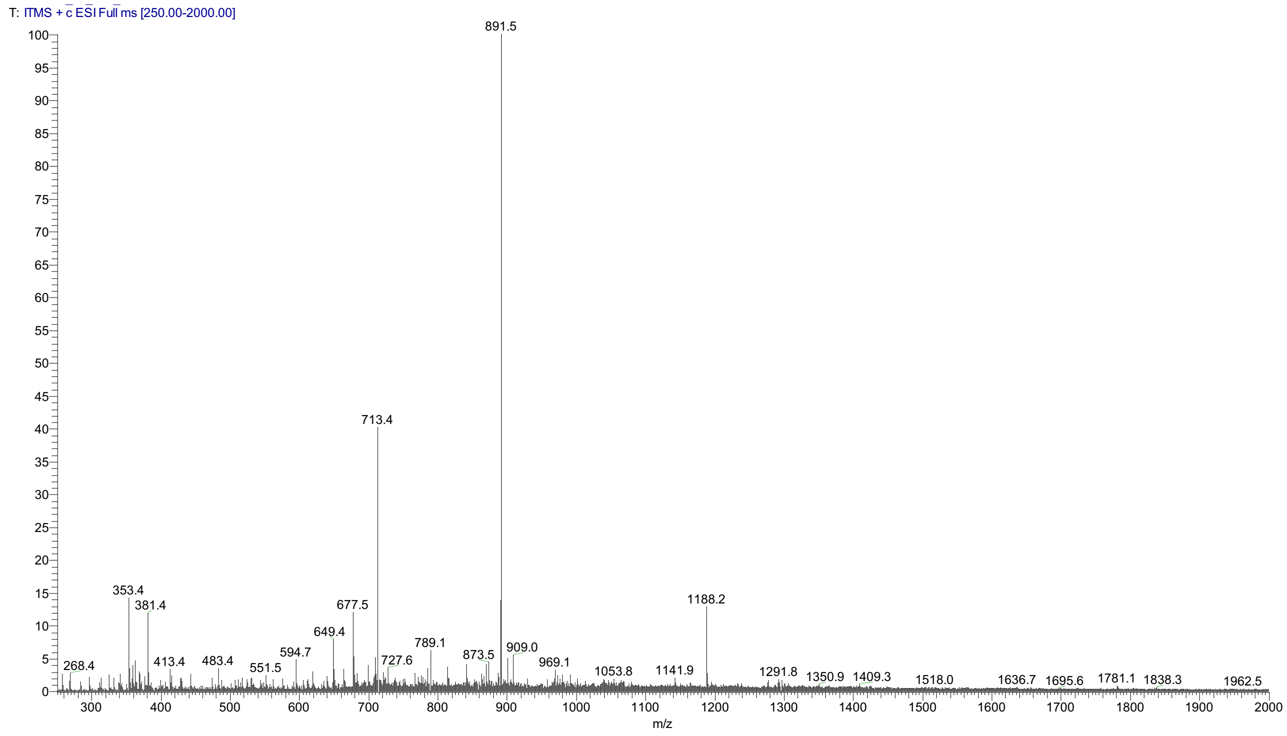

Figure S13. ESI-MS of peptide **P3**. Calculated mass: $[M+3H]^{+}/3$= 1188.1; $[M+4H]^{4+}/4$= 891.3. Found mass: $[M+3H]^{3+}/3$=1188.2; $[M+4H]^{4+}/4$=891.5.

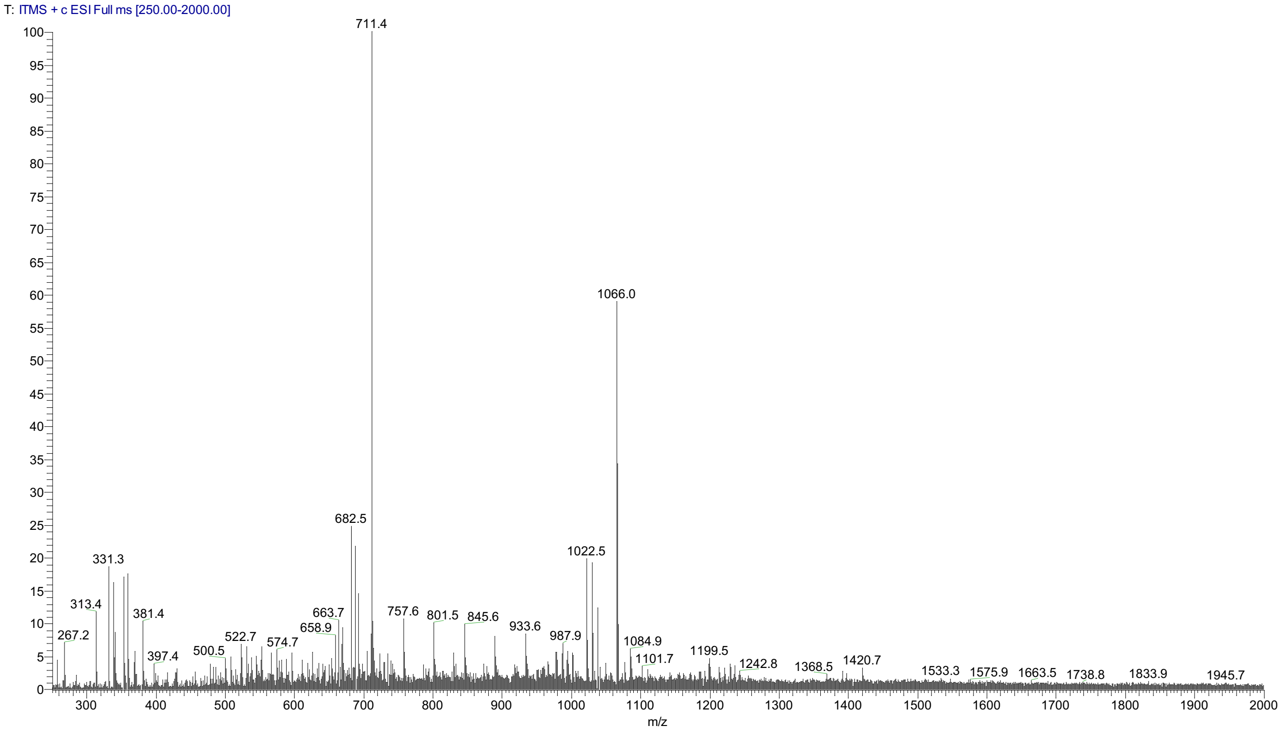

Figure S14. ESI-MS of peptide **P2-t**. Calculated mass: $[M+2H]^+/2$= 1066.3; $[M+3H]^+/3$= 711.2. Found mass: $[M+2H]^+/2$= 1066.1; $[M+3H]^+/3$= 711.4.

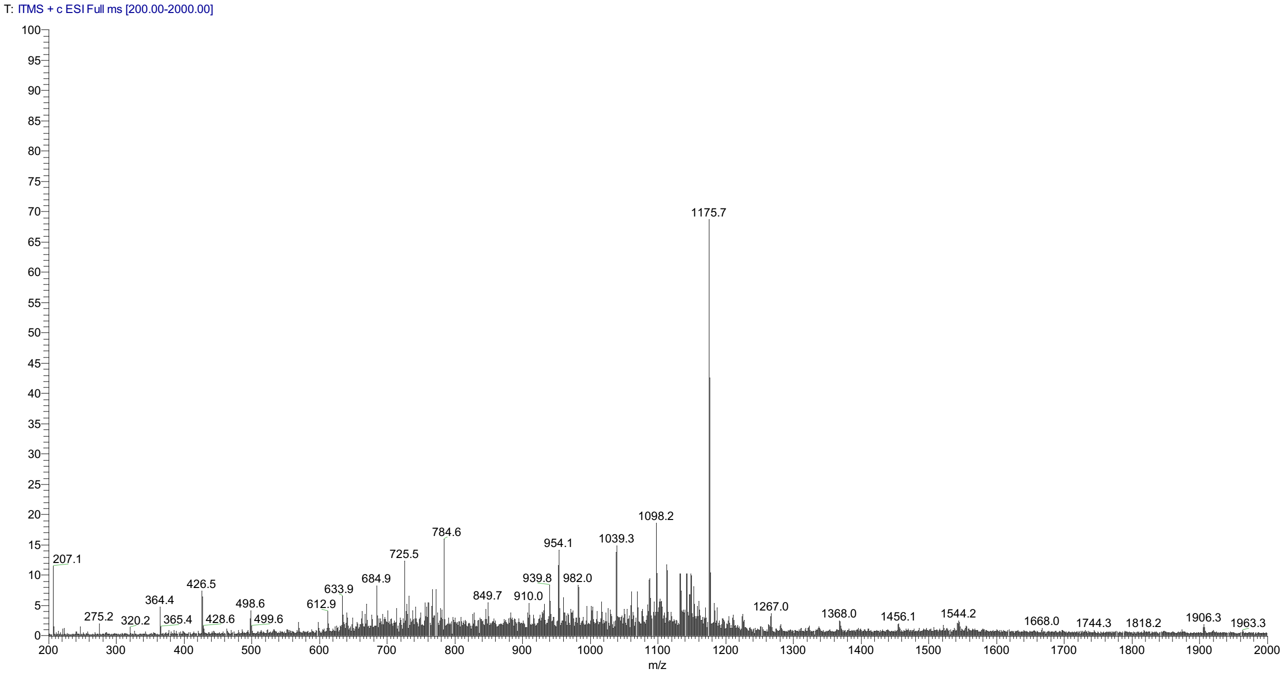

Figure S15. ESI-MS of peptide **P2-TMZ**. Calculated mass: $[M+2H]^+/2$= 1175.5; $[M+3H]^+/3$= 783.9. Found mass: $[M+2H]^+/2$= 1175.7; $[M+3H]^+/3$= 784.6.

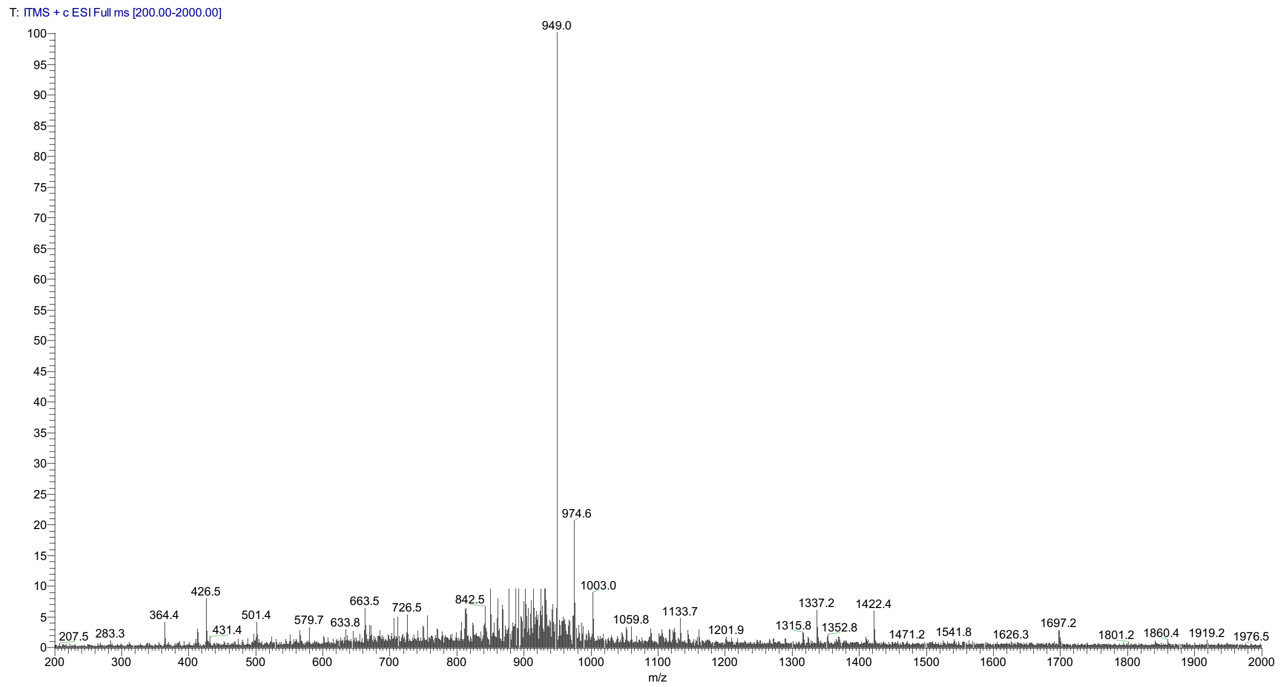

Figure S16. ESI-MS of peptide **P2-BrP**. Calculated mass: $[M+3H]^+/3$= 949.3. Found mass: $[M+3H]^+/3$= 949.0.
